# Decoding Atrial Fibrillation Heterogeneity: A Nationwide Phenome-Wide Association Study in England

**DOI:** 10.1016/j.jacadv.2026.103352

**Published:** 2026-10-22

**Authors:** Hiroyuki Yoshimura, Sheng-Chia Chung, Gregory Y.H. Lip, A. Floriaan Schmidt, Rui Providencia

**Affiliations:** a Institute of Health Informatics Research, University College London, London, United Kingdom; b Liverpool Centre for Cardiovascular Science at University of Liverpool, Liverpool John Moores University and Liverpool Heart & Chest Hospital, Liverpool, United Kingdom; c Danish Center for Clinical Health Services Research, Department of Clinical Medicine, Aalborg University, Aalborg, Denmark; d Department of Cardiology, Lipidology and Internal Medicine with Intensive Coronary Care Unit, Medical University of Bialystok, Bialystok, Poland; e Institute of Cardiovascular Science, Faculty of Population Health, University College London, London, United Kingdom; f UCL British Heart Foundation Center of Excellence, London, United Kingdom; g Department of Cardiology, Amsterdam Cardiovascular Sciences, Amsterdam University Medical Centres, University of Amsterdam, Amsterdam UMC, the Netherlands; h Barts Heart Centre, Barts Health NHS Trust, London, United Kingdom

**Keywords:** agnostic, arrhythmia, comorbidities, Mendelian randomization, precision medicine, sex differences

## Abstract

**Background:**

Age and sex influence atrial fibrillation (AF) prevalence and outcomes, yet comprehensive data on sex- and age-specific health care utilization in patients newly diagnosed with AF remain limited.

**Objectives:**

We aimed to address this gap using a phenome-wide association study (Phe-WAS) approach.

**Methods:**

We conducted a retrospective cohort study using nationwide Clinical Practice Research Datalink data linked to Hospital Episodes Statistics. Adults diagnosed with AF between 1998 and 2016 (n = 199,433) were matched 1:1 by age and sex to non-AF control subjects. Primary and secondary care interactions 5 years post diagnosis were analyzed using Phe-WAS with Bonferroni-adjusted significance thresholds. Logistic regression estimated odds ratios for conditions in AF vs control groups, stratified by sex and age. Associations were assessed for novelty through literature review and causality using published Mendelian randomization studies.

**Results:**

Patients with AF had higher rates of cardiovascular and noncardiovascular conditions than control subjects, with marked sex- and age-specific patterns. Women had more bleeding complications, pneumonia, *Clostridium difficile* enterocolitis, cholangitis, and electrolyte disturbances, whereas men had higher rates of alcohol-related disorders, ventricular arrhythmias, cardiac arrest, and cardiomyopathy. Younger patients with AF (<50 years) more often had alcohol-related mental and behavioral disorders, gout, and dilated cardiomyopathy, while older patients (>60 years) more often had heart failure, ischemic stroke, renal failure, and respiratory infections. Of 51 phenotypes more common in AF, 28 were previously reported, and 23 were potentially novel. Mendelian randomization analyses suggested AF was causal for 19 associations.

**Conclusions:**

This Phe-WAS demonstrates the broad spectrum of health care interactions in AF beyond traditional cardiovascular complications.

Atrial fibrillation (AF) is the most common type of cardiac arrhythmia, and its worldwide prevalence, incidence, and mortality are rapidly increasing.^1^ A 2023 Global Burden of Disease study estimated a total of 59.0 million AF cases and 378,000 deaths from the disorder globally, representing significant increases of 111% and 223%, respectively, compared with the 1990 figures.^2^

The age and sex disparities in AF prevalence are well-established: Men have a higher prevalence under 70 years of age, prevalence is similar between 70 and 74, and women have a higher prevalence over 75.^3^ In the United Kingdom, similar trends have been observed, with higher AF incidence and mortality in men younger than 70 years and comparable rates in older individuals.^4^ Furthermore, previous cohort studies^5^, ^6^, ^7^ and patient registries^8^^,^^9^ indicate important differences in outcomes among patients with AF based on age and sex.

These findings underscore the need to consider age and sex in AF management. However, there is a knowledge gap regarding sex-specific, age-related health care utilization patterns for individuals newly diagnosed with AF. This is important given the impact of AF on health care costs, with the main determinant being hospitalizations.^10^

Genome-wide association studies have been at the forefront of hypothesis-free association discovery, which occasionally results in the identification of significant but unexpected relationships.^11^ Studies that replicate the same methods using clinical data (ie, diagnostic codes), named Phenome-Wide Association Studies (Phe-WAS), have the ability to describe the entire range of illnesses and consequences that occur within patient groups.^12^

A better understanding of health care contacts in the AF population for particular age-strata and sex groups represents a great opportunity to further clarify this heterogeneity conundrum in the era of precision medicine, where the goal is to use a tailored therapy to address the needs of specific patients. This study uses a Phe-WAS approach to determine the most frequent reasons for health care interaction among individuals with AF compared to those without, stratified by sex and age. The findings will help to develop sex- and age-specific care plans, potentially preventing or delaying the onset of AF-related comorbidities in at-risk patient groups.

## Methods

### Data sources, study population, and design

As of 2018, 7,998,501 patients in the United Kingdom were part of the Clinical Practice Research Datalink (CPRD), which was founded in 1987,^13^ and contains linked data from primary care consultations, hospitals (Hospital Episodes Statistics, HES), national cancer registries (National Cancer Intelligence Network), and death registries (Office for National Statistics).^14^^,^^15^ These data are generally representative of the age, gender, and geographic distribution of the UK population^10^ and showed high quality and completeness of clinical information recorded.^14^, ^15^, ^16^ The CPRD has received ethical approval (REC: 21/EM/0265). The present study was approved by the Medicines and Healthcare products Regulatory Agency Independent Scientific Advisory Committee (17_205).

This retrospective cohort study included individuals aged 18 years or older, who were registered in the primary care practice for at least 1 year between January 1, 1998, and May 31, 2016. Individuals with a prior history of AF before the study entry were excluded. AF was identified using the International Classification of Diseases, tenth revision (ICD-10) as I48 from HES and Read codes from CPRD (Supplemental Table 1).

A matched case-control study was implemented to compare the most frequent problems and comorbidities in patients with AF to those in a control group. For each patient with AF, the reasons for General Practitioner visits (from CPRD) and primary diagnoses for hospital admissions (from HES) were identified in the 5 years following their initial diagnosis. Codes related to the AF diagnosis were excluded. For comparison, the top conditions associated with post–index-date clinical visits were also determined for the matched control group. The control subjects were individuals who had health care interactions but no history of AF and were individually matched 1:1 to patients with AF by age and sex. The matching age was determined at the time of the respective health care interaction for each pair. All analyses were stratified by sex and across defined age strata (<50, 50-59, 60-69, 70-79, 80-89, and ≥90 years).

A secondary analysis was performed to assess phenotypes associated with AF and not dependent on the presence of ischemic heart disease. For this, we compared all patients with AF but without ischemic heart disease at baseline vs 1:1 age- and sex-matched control subjects with ischemic heart disease and no known AF at baseline.

### Statistical analyses and literature review

Baseline characteristics were summarized for patients with AF and for non-AF control subjects. Categorical data were reported as counts and percentages, while continuous data were reported as means with SD.

We performed a time-trend analysis of oral anticoagulant utilization and the rates of ischemic stroke, pulmonary embolism, and major bleeding between 2000 and 2015. The electronic health record phenotypes used are described in detail in Supplemental Table 1.

Using binary logistic regression, we estimated ORs and 95% CIs for the occurrence of specific health care interactions in the AF group compared to the non-AF group. Furthermore, we conducted a Phe-WAS to determine the top 100 conditions in patients with AF when compared to sex- and age-matched control subjects, ranked in descending order of OR based on the previously described methodology.^17^

Given the large number of logistic regressions performed simultaneously to identify causes for hospitalization or primary care visits, the Bonferroni correction was applied to control the family-wise error rate and minimize the risk of type I errors.

Primary Phe-WAS analyses were conducted using stricter Bonferroni-adjusted thresholds reflecting the total number of codes tested within each data set: an adjusted *P* < 5 × 10^−7^ (based on 100,000 Read codes, mapped to medcodes, from CPRD) for primary care visits,^18^ and *P* < 3.78 × 10^−6^ for hospitalizations (based on 13,232 ICD-10 codes from HES^19^). Secondary exploratory analyses adjusting only for the top 100 associations with AF (ie, the outputs used for this manuscript) were performed for identifying other potential associations and using a significance threshold of *P* < 0.0005 (ie, 0.05/100).

A time-to-event analysis was not performed because the study's objective was to assess the cumulative frequency of specific causes for health care interactions over a prespecified 5-year period. This timeframe accounts for the high 5-year mortality rate observed in patients with new-onset AF.^4^ The study design is analogous to a genome-wide association study, which captures a static “snapshot” of exposure rather than tracking events over time.

Manhattan plots were generated to visualize results across different population strata. On the x-axis, electronic health record codes (including ICD-10 and medcodes) were plotted against their corresponding log *P* values on the y-axis.

To identify the organ systems associated with health care contacts in different population groups, we first converted the identified “hits” that met a multiple-adjusted *P* value threshold into higher-level clinical phenotypes. For example, codes for both non-ST-elevation and ST-elevation myocardial infarction were grouped under the phenotype “myocardial infarction”. For both primary and secondary care data, we mapped electronic health record codes to their corresponding ICD-10 chapters. We then plotted the number of phenotypes for each ICD-10 chapter.

Finally, for all identified associations meeting the multiple adjusted *P* value threshold, we conducted a literature review of MedLine to assess novelty and investigated causality using previously published Mendelian randomization studies (MR). MR is a causal inference method that uses genetic variants as instrumental variables to investigate the causal effect of an exposure on a health outcome using observational data. This approach is sometimes described as analogous to a “*natural randomized controlled trial*” because genetic variants are randomly assigned at conception, similar to how interventions are randomly assigned in a clinical trial. This natural randomization helps ensure that the genetic instrumental variable is not associated with the confounders that undermine conventional observational research. MR studies have demonstrated that individuals who inherited the gene variants for lower low-density lipoprotein cholesterol levels had a significantly lower risk of cardiovascular events and higher risk of diabetes,^20^ proving the causality in the previously described association.

Our searches used structured expressions, such as ((“atrial fibrillation” AND “myocardial infarction” AND (“nationwide” OR “case-control” OR “observational”)) to identify previous reports on the associations, and ((“mendelian randomization” OR “genetically predicted”) AND “atrial fibrillation” AND “myocardial infarction”)) to identify MR studies with AF as the exposure.

All analyses were performed using Statistical Analysis System (version 9.4) and R (version 4.5.1).

## Results

From a cohort of 5,557,405 eligible individuals, 199,433 were newly diagnosed with AF between January 1, 1998, and May 31, 2016. The average age of diagnosis was 75.7 years (SD: ±12.7), and half of the patients were women (50.2%). A comparison group of 199,433 individuals was matched for age and sex (Table 1).

**Table 1:** Characteristics of Patients With Newly Diagnosed Atrial Fibrillation at Study Entry

	All AF Patients n = 1,99 ,433	Non-AF Control Subjects n = 1,99 ,433	AF Stratified by Sex		AF Stratified by Age at Diagnosis					
			Men n = 99,281	Women n = 100,152	<50 n = 8,086	50-59 n = 12,749	60-69 n = 31,005	70-79 n = 58,992	80-89 n = 67,364	≥90 n = 21,307
Age at diagnosis (y)	75.7 (12.7)	75.7 (12.7)	72.7 (13.1)	78.9 (11.5)	—	—	—	—	—	—
Women	100,152 (50.2%)	100,152 (50.2%)	—	—	2,302 (28.5%)	3,880 (30.4%)	11,227 (36.2%)	27,608 (46.9%)	39,189 (59.1%)	15,316 (71.9%)
Smoking	86,207 (43.2%)	32,130 (16.1%)	53,004 (53.4%)	33,203 (33.2%)	3,129 (38.7%)	5,970 (46.8%)	16,322 (52.6%)	27,284 (47.2%)	26,662 (39.6%)	6,300 (29.6%)
CHA_2_DS_2_-VASc: ≥1♂ or ≥2♀	173,354 (86.9%)	171,889 (86.2%)	82,484 (83.1%)	90,870 (90.7%)	1,038 (12.8%)	3,199 (25.1%)	13,076 (42.2%)	49,143 (83.4%)	67,116 (99.6%)	21,307 (100%)
Comorbidities										
Hypertension	99,644 (50.0%)	45,182 (22.7%)	46,309 (46.6%)	53,335 (53.3%)	1,317 (16.3%)	4,226 (33.1%)	13,561 (43.7%)	30,112 (51.1%)	38,129 (56.6%)	12,299 (57.7%)
Diabetes	31,099 (15.5%)	12,487 (6.3%)	17,064 (17.2%)	13,945 (13.9%)	537 (6.6%)	1,670 (13.1%)	5,246 (16.9%)	10,814 (18.4%)	10,331 (15.3%)	2,411 (11.3%)
Valvular disease	20,467 (10.3%)	1941 (1%)	9,870 (9.9%)	10,597 (10.6%)	694 (8.6%)	1,119 (8.8%)	3,027 (9.8%)	6,317 (10.7%)	7,097 (10.5%)	2,213 (10.4%)
Angina	37,846 (19.0%)	14,858 (7.5%)	20,854 (21.0%)	16,992 (17.0%)	323 (4.0%)	1,434 (11.2%)	5,370 (17.3%)	12,802 (21.7%)	14,054 (20.9%)	3,836 (18.1%)
Ischemic heart disease	33,358 (16.7%)	10,349 (5.2%)	19,897 (20.0%)	13,461 (13.4%)	455 (5.6%)	1,621 (12.7%)	5,096 (16.4%)	11,060 (18.8%)	11,801 (17.5%)	3,325 (15.6%)
Heart failure	41,925 (21.0%)	791 (0.4%)	20,236 (20.4%)	21,689 (21.7%)	628 (7.8%)	1,543 (11.4%)	4,445 (15.3%)	11,358 (19.3%)	17,141 (25.4%)	6,900 (32.4%)
Stroke	23,208 (11.6%)	7,363 (3.7%)	10,897 (11.0%)	12,311 (12.3%)	202 (2.5%)	594 (4.7%)	2,424 (7.8%)	6,881 (11.7%)	9,821 (14.6%)	3,286 (15.4%)
Asthma	30,252 (15.2%)	12,828 (6.4%)	14,059 (14.2%)	16,193 (16.2%)	1,368 (16.9%)	1982 (15.5%)	5,065 (16.3%)	9,703 (16.5%)	9,784 (14.5%)	2,350 (11.0%)
Hyperthyroidism	4,825 (2.4%)	1932 (1%)	1,028 (1.0%)	3,797 (3.8%)	188 (2.3%)	274 (2.1%)	649 (2.1%)	1,384 (2.3%)	1812 (2.7%)	518 (2.4%)
Cancer	38,976 (19.5%)	20,306 (10.2%)	20,263 (20.4%)	18,713 (18.7%)	289 (3.6%)	1,061 (8.3%)	4,501 (14.5%)	11,486 (19.5%)	15,981 (23.7%)	5,658 (26.6%)
Chronic kidney disease	40,362 (20.2%)	4,121 (2.1%)	18,985 (19.1%)	21,377 (21.3%)	474 (5.9%)	1,072 (8.4%)	3,862 (12.5%)	10,795 (18.3%)	17,435 (25.9%)	6,724 (31.6%)
Chronic obstructive pulmonary disease	25,795 (12.9%)	8,207 (4.1%)	14,182 (14.3%)	11,613 (11.6%)	174 (2.2%)	813 (6.4%)	3,841 (12.4%)	8,972 (15.2%)	9,786 (14.5%)	2,209 (10.4%)
Dementia	12,254 (6.1%)	4,674 (2.3%)	4,354 (4.4%)	7,900 (7.9%)	12 (0.1%)	28 (0.2%)	217 (0.7%)	1862 (3.2%)	6,646 (9.9%)	3,489 (16.4%)
Two or more comorbidities^a^	36,176 (18.1%)	118,661 (59.5%)	57,925 (58.3%)	60,736 (60.6%)	704 (8.7%)	2,421 (19.0%)	9,111 (29.4%)	23,114 (39.2%)	30,398 (45.1%)	10,148 (47.6%)

Data are presented as mean (SD) or n (%).

AF = atrial fibrillation.

a Number of comorbidities refers to any of the 13 conditions listed.

Upon the initial AF diagnosis, men had a higher prevalence of smoking and various comorbidities, including diabetes, coronary heart disease, cancer, and chronic obstructive pulmonary disease. By comparison, women were more likely to have hypertension, heart failure, asthma, hyperthyroidism, chronic kidney disease, and dementia.

Individuals diagnosed with AF before age 50 were more often men and had a significantly lower prevalence of hypertension than older patients with AF. While the sex distribution was equal across the overall cohort, the proportion of women with AF rose with age. By age 80 years or above, women made up a larger share of new-onset AF diagnoses. Advancing age was also associated with a greater number of comorbidities per patient.

At 5 years, mortality was 42.7% (95% CI: 42.4%-42.9%) among patients with AF and 26.0% (95% CI: 25.8%-26.2%) among matched control subjects. A total of 31.0% of patients with AF and 59.8% of control subjects completed the 5-year follow-up.

During the study period, an increase in the uptake of oral anticoagulants and in the rate of ischemic stroke and major bleeding was observed (Supplemental Figure 1).

### Primary analyses

The results discussed later illustrate findings based on the prespecified, multiply adjusted *P* value thresholds of *P* < 5 × 10^−7^ and *P* < 3.78 × 10^−6^ for primary and secondary care, respectively. For a more detailed look at the 100 most frequent associations for each stratum, see Supplemental Data file.

### Health care contacts stratified by sex

Compared with matched control subjects, women with AF had a higher frequency of encounters for 17 reasons in primary care and hospitalizations for 27 conditions in secondary care. In secondary care, significantly higher odds ratios were found for “congestive heart failure” (OR: 3.64; 95% CI: 3.48-4.12; *P* = 2.1 × 10^−258^), “lobar pneumonia, unspecified” (OR: 3.74; 95% CI: 3.51-4.25; *P* = 1.39 × 10^−212^), “cerebral infarction, unspecified” (OR: 2.63; 95% CI: 2.50-2.93; *P* = 6.85 × 10^−148^), “tendency to fall, not elsewhere classified” (OR: 16.56; 95% CI: 9.88-28.02; *P* = 6.73 × 10^−56^), “acute renal failure, unspecified” (OR: 3.75; 95% CI: 3.12-4.57; *P* = 6.51 × 10^−52^), and “aortic (valve) stenosis” (OR: 4.32; 95% CI: 3.36-5.61; *P* = 8.95 × 10^−37^), among others. In primary care, notable associations include “acute exacerbation of chronic obstructive airways disease” (OR: 5.57; 95% CI: 4.27-7.36; *P* = 2.26 × 10^−48^), “acute non-ST-segment elevation myocardial infarction” (OR: 13.91; 95% CI: 8.41-23.20; *P* = 7.10 × 10^−48^), “mitral regurgitation” (OR: 9.25; 95% CI: 6.20-13.92; *P* = 7.29 × 10^−45^), and “pulmonary embolism” (OR: 5.43; 95% CI: 3.97-7.50; *P* = 1.63 × 10^−34^). A comprehensive summary of these findings is available in Tables 2 and 3 and is visualized in the Manhattan plots (Figure 1A and Supplemental Figures 2 and 3).

**Table 2:** Top Reasons for Hospitalization in Atrial Fibrillation Patients, Compared to Control Subjects, Stratified by Sex

Men (n = 99,281)						Women (n = 100,152)					
Reason for Hospitalization	n AF	n Control Subjects	OR	95% CI	*P*	Reason for Hospitalization	n AF	n Control Subjects	OR	95% CI	*P*
Cerebral infarction due to embolism of cerebral artery	110	3	36.6	11.7-115.6	7.4E-10	Gastroenteritis and colitis of unspecified origin	221	3	73.5	23.6-230.7	1.4E-13
Dilated cardiomyopathy	169	9	18.8	9.6-36.8	2.1E-39	Cerebral infarction due to embolism of cerebral artery	156	3	51.9	16.6-163.2	1.2E-11
Mitral (valve) insufficiency	201	12	16.7	9.4-30.1	2.0E-45	Disorders of both mitral and tricuspid valves	52	3	17.3	5.4-55.3	1.5E-06
Chronic kidney disease, stage 5	109	9	12.1	6.1-23.9	6.3E-13	Mitral (valve) insufficiency	154	9	17.1	8.8-33.6	2.2E-16
Mental and behavioral disorders due to the use of alcohol	109	9	11.8	6.0-23.3	1.2E-12	Tendency to fall, not elsewhere classified	249	15	16.6	9.9-28.0	6.7E-56
Cardiac arrest with successful resuscitation	53	6	8.8	3.8-20.6	4.2E-07	Chronic kidney disease, stage 5	58	6	9.7	4.2-22.4	1.2E-07
Tendency to fall, not elsewhere classified	162	21	7.7	4.9-12.2	3.4E-28	Acute and subacute infective endocarditis	50	6	8.3	3.6-19.4	9.2E-07
Supraventricular tachycardia	314	45	7.0	5.1-9.6	7.4E-51	Cerebral infarction due to thrombosis of cerebral artery	121	15	8.1	4.7-13.8	2.4E-14
Ventricular tachycardia	235	36	6.5	4.6-9.3	5.1E-37	Cerebral infarction due to unspecified occlusion or stenosis	484	60	8.0	6.2-10.6	1.4E-83
Cerebral infarction due to thrombosis of cerebral artery	95	15	6.3	3.7-10.9	3.0E-11	Thyrotoxicosis, unspecified	60	12	5.0	2.7-9.3	3.6E-07
Aortic (valve) insufficiency	53	9	5.9	2.9-11.9	8.7E-07	Ventricular tachycardia	68	15	4.5	2.6-7.9	1.2E-07
Gastroenteritis and colitis of unspecified origin	140	27	5.2	3.4-7.8	4.7E-15	Hyperkalemia	67	15	4.5	2.5-7.8	1.6E-07
Acute and subacute infective endocarditis	105	21	5.0	3.1-8.0	1.6E-11	Sick sinus syndrome	159	36	4.4	3.1-6.4	8.8E-16
Disorders of both mitral and aortic valves	59	12	4.9	2.6-9.2	4.9E-07	Supraventricular tachycardia	316	72	4.4	3.4-5.7	1.3E-37
Cerebral infarction due to unspecified occlusion or stenosis	286	66	4.3	3.3-5.7	8.1E-34	Aortic (valve) stenosis	312	72	4.3	3.4-5.6	8.9E-37
Barrett's esophagus	59	15	3.9	2.2-6.9	2.2E-06	Other specified abnormal findings of blood chemistry	58	15	3.9	2.2-6.8	3.0E-06
Congestive heart failure	2,396	606	3.9	3.7-4.4	1.6E-253	Acute renal failure, unspecified	508	135	3.8	3.1-4.6	6.5E-52
Aortic (valve) stenosis	319	84	3.8	3.0-4.8	2.2E-33	Lobar pneumonia, unspecified	2088	549	3.7	3.5-4.3	1.4E-212
Precordial pain	115	33	3.5	2.4-5.1	2.5E-10	Congestive heart failure	2,596	699	3.6	3.5-4.1	2.1E-258
Palpitations	95	30	3.2	2.1-4.8	3.7E-08	Heart failure, unspecified	224	66	3.4	2.6-4.5	2.6E-21
Lobar pneumonia, unspecified	1981	690	2.8	2.7-3.2	3.5E-145	Diffuse large B-cell lymphoma	70	21	3.3	2.1-5.4	1.3E-06
Acute myeloblastic leukemia	90	33	2.7	1.8-4.1	8.1E-07	Respiratory failure, unspecified	97	30	3.2	2.2-4.9	1.9E-08
Polyp of colon	142	54	2.6	1.9-3.6	1.4E-09	Precordial pain	84	27	3.1	2.0-4.8	2.9E-07
Pleural effusion, not elsewhere classified	496	189	2.6	2.2-3.1	9.3E-33	Embolism and thrombosis of arteries of lower extremities	129	42	3.1	2.2-4.4	2.6E-10
Acute renal failure, unspecified	506	198	2.6	2.2-3.0	4.6E-32	End-stage renal disease	98	33	3.0	2.0-4.4	6.3E-08
Sick sinus syndrome	96	39	2.5	1.7-3.6	2.1E-06	Enterocolitis due to *Clostridium difficile*	288	99	2.9	2.3-3.7	1.5E-22
						Cerebral infarction, unspecified	2,242	840	2.6	2.5-2.9	6.9E-148

All results pass the threshold for multiple adjusted comparisons of *P* < 0.0000038 (ie, *P* < 3.8E-06).

AF = atrial fibrillation.

**Table 3:** Top Reasons for Primary Care Consultations in Atrial Fibrillation Patients, Compared to Control Subjects, Stratified by Sex

Men (n = 99,281)						Women (n = 100,152)					
Reason for Primary Care Visit	n AF	n Control Subjects	OR	95% CI	*P*	Reason for Primary Care Visit	n AF	n Control Subjects	OR	95% CI	*P*
Mitral regurgitation	242	20	12.1	7.7-19.1	1.2E-49	Acute non-ST-segment elevation myocardial infarction	223	16	13.9	8.4-23.2	7.1E-48
Primary pulmonary hypertension	64	6	10.7	4.6-24.7	2.9E-08	Acute coronary syndrome	94	7	13.4	6.2-28.9	3.3E-11
Acute non-ST-segment elevation myocardial infarction	229	28	8.2	5.5-12.1	1.9E-40	Mitral regurgitation	241	26	9.3	6.2-13.9	7.3E-45
Sepsis	81	10	8.1	4.2-15.6	4.3E-10	Sepsis	82	12	6.8	3.7-12.5	5.0E-10
Thyrotoxicosis	91	13	7.0	3.9-12.5	5.2E-11	Acute renal failure	70	12	5.8	3.2-10.8	1.6E-08
Supraventricular tachycardia NOS	92	14	6.6	3.8-11.5	5.2E-11	Pneumonia due to unspecified organism	496	87	5.7	4.6-7.2	1.1E-70
Heart failure	367	59	6.2	4.7-8.2	1.6E-55	Acute exacerbation of chronic obstructive airways disease	341	61	5.6	4.3-7.4	2.3E-48
Acute renal failure	60	11	5.5	2.9-10.4	3.0E-07	Cerebral infarction NOS	137	25	5.5	3.6-8.4	5.1E-15
Pleural effusion NOS	258	52	5.0	3.7-6.7	5.1E-34	Pulmonary embolism	245	45	5.4	4.0-7.5	1.6E-34
Pulmonary embolism	191	43	4.4	3.2-6.2	1.8E-23	Pleural effusion NOS	155	29	5.3	3.6-8.0	2.2E-16
Acute coronary syndrome	62	14	4.4	2.5-7.9	4.9E-07	Cardiac failure	67	14	4.8	2.7-8.5	9.9E-08
Coronary artery disease	118	28	4.2	2.8-6.4	7.6E-12	Septicemia	87	19	4.6	2.8-7.5	1.9E-09
Congestive cardiac failure	402	99	4.1	3.3-5.1	2.0E-44	CVA—cerebral artery occlusion	184	41	4.5	3.2-6.3	7.6E-23
Chronic obstructive pulmonary disease with acute exacerbation, unspecified	113	29	3.9	2.6-5.9	6.3E-11	Lower respiratory tract infection	251	61	4.1	3.1-5.5	2.2E-29
Acute exacerbation of chronic obstructive airways disease	424	109	3.9	3.2-4.8	5.8E-45	Thyrotoxicosis	151	37	4.1	2.9-5.9	1.7E-14
CVA—cerebral artery occlusion	148	41	3.6	2.6-5.1	3.4E-13	Heart failure	311	80	3.9	3.1-5.0	2.6E-33
						Renal failure unspecified	86	23	3.7	2.4-5.9	1.9E-08

All results pass the threshold for multiple adjusted comparisons of *P* < 0.0000005 (ie, *P* < 5E-07).

AF = atrial fibrillation; CVA = cardiovascular accident; NOS = not otherwise specified.

**Figure 1:**
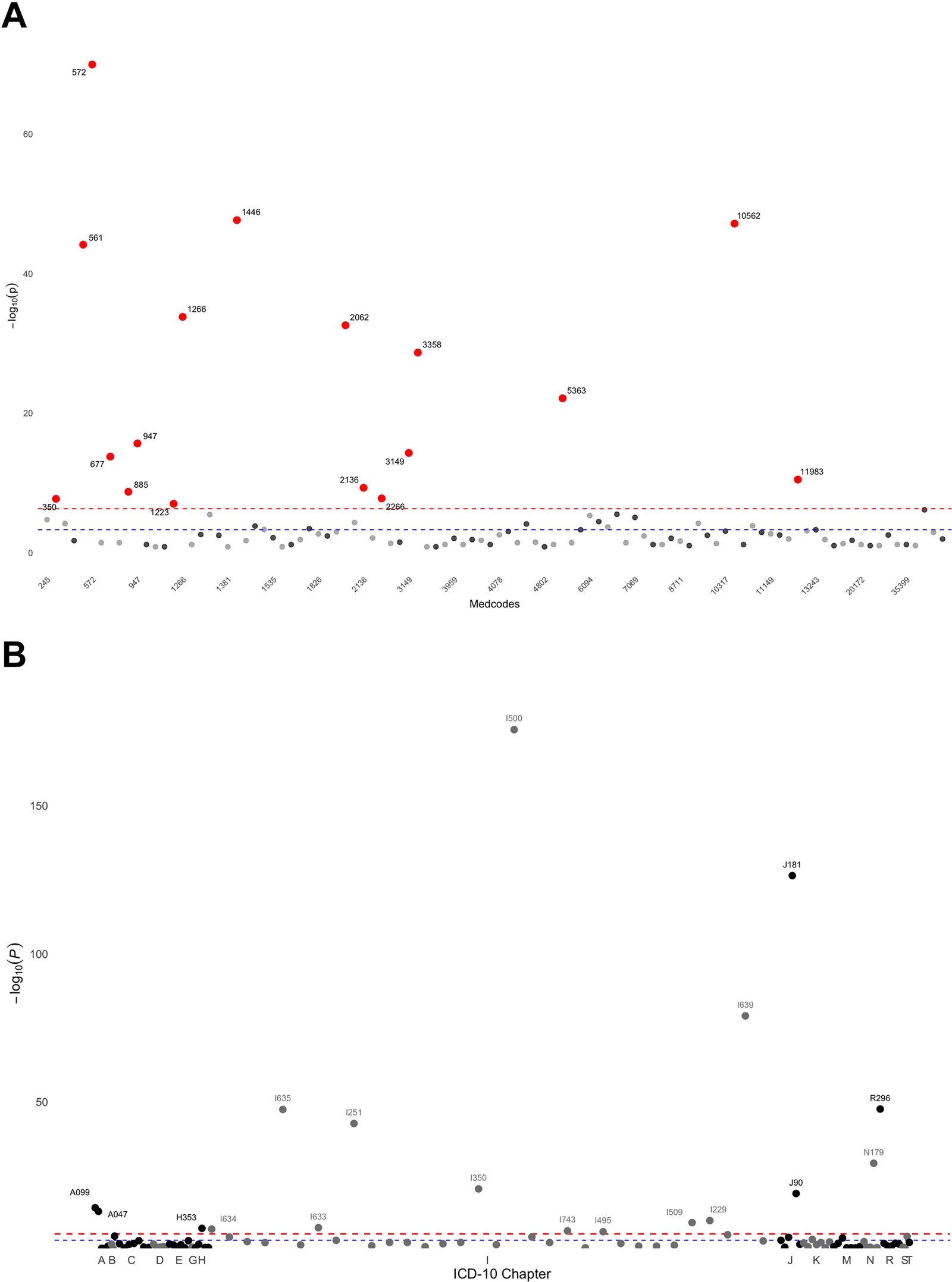
Manhattan Plots Showing Primary Care Interactions for Women With AF and Secondary Care Interactions for Patients With AF Aged 80 to 89 Years Manhattan plots showing medcodes more frequently identified in women with AF (A) and ICD-10 codes in patients with AF aged 80 to 89 (B). Panel A shows the medcodes meeting the significance threshold for the primary analyses (*P* < 5 × 10^−7^), highlighted in red: 572 = pneumonia due to unspecified organism; 1,446 = acute exacerbation of chronic obstructive airways disease; 10,562 = acute non-ST-segment elevation myocardial infarction; 561 = mitral regurgitation; 1,266 = pulmonary embolism; 2062 = heart failure; 3,358 = lower respiratory tract infection; 5,363 = CVA, cerebral artery occlusion; 947 = pleural effusion NOS; 3,149 = cerebral infarction NOS; 677 = thyrotoxicosis; 11,983 = acute coronary syndrome; 2,136 = sepsis; 885 = septicemia; 2,266 = acute renal failure; 350 = renal failure unspecified; 1,223 = cardiac failure. Panel B shows the ICD-10 codes across the different chapters meeting the significance threshold for the primary analyses (*P* < 3.78 × 10^−6^): I500 = congestive heart failure; J181 = lobar pneumonia, unspecified; I639 = cerebral infarction, unspecified; R296 = tendency to fall, not elsewhere classified; I635 = cerebral infarction due to unspecified occlusion or stenosis; I251 = atherosclerotic heart disease; N179 = acute renal failure, unspecified; I350 = aortic (valve) stenosis; J90 = pleural effusion, not elsewhere classified; A099 = gastroenteritis and colitis of unspecified origin; A047 = enterocolitis due to *Clostridium difficile*; I229 = subsequent myocardial infarction of unspecified site; I509 = heart failure, unspecified; I633 = cerebral infarction due to thrombosis of cerebral artery; H353 = degeneration of macula and posterior pole; I634 = cerebral infarction due to embolism of cerebral artery; I743 = embolism and thrombosis of arteries of lower extremities; I495 = sick sinus syndrome. AF = atrial fibrillation; CVA = cardiovascular accident; ICD-10 = International Classification of Diseases, tenth revision; NOS = not otherwise specified.

Relative to their matched control subjects, men with AF significantly more often had primary care visits for 16 specific reasons and secondary care admissions for 26 diagnoses (Supplemental Figures 2 and 3). For secondary care, significant associations included “congestive heart failure” (OR: 3.88; 95% CI: 3.68-4.40; *P* = 1.57 × 10^−253^), “lobar pneumonia, unspecified” (OR: 2.83; 95% CI: 2.67-3.17; *P* = 3.52 × 10^−145^), “supraventricular tachycardia” (OR: 6.96; 95% CI: 5.12-9.56; *P* = 7.41 × 10^−51^), “mitral (valve) insufficiency” (OR: 16.72; 95% CI: 9.37-30.05; *P* = 1.96 × 10^−45^) in secondary care, “dilated cardiomyopathy” (OR: 18.75; 95% CI: 9.62-36.78; *P* = 2.08 × 10^−39^), “ventricular tachycardia” (OR: 6.51; 95% CI: 4.61-9.29; *P* = 5.12 × 10^−37^), and “cerebral infarction due to unspecified occlusion or stenosis” (OR: 4.32; 95% CI: 3.32-5.68; *P* = 8.06 × 10^−34^). In primary care, notable findings included “mitral regurgitation” (OR: 12.07; 95% CI: 7.69-19.14; *P* = 1.18 × 10^−49^), “acute exacerbation of chronic obstructive pulmonary disease” (OR: 3.88; 95% CI: 3.16-4.82; *P* = 5.83 × 10^−45^), and “non-ST elevation myocardial infarction” (OR: 8.16; 95% CI: 5.53-12.13; *P* = 1.90 × 10^−40^).

An analysis of organ systems revealed that the circulatory system was the most commonly associated system, with 24 associations for men and 22 for women (Figure 2). The respiratory system ranked second, with 5 associations for men and 6 for women, with the genitourinary system being the third most frequent, with 3 associations for men and 5 for women.

**Figure 2:**
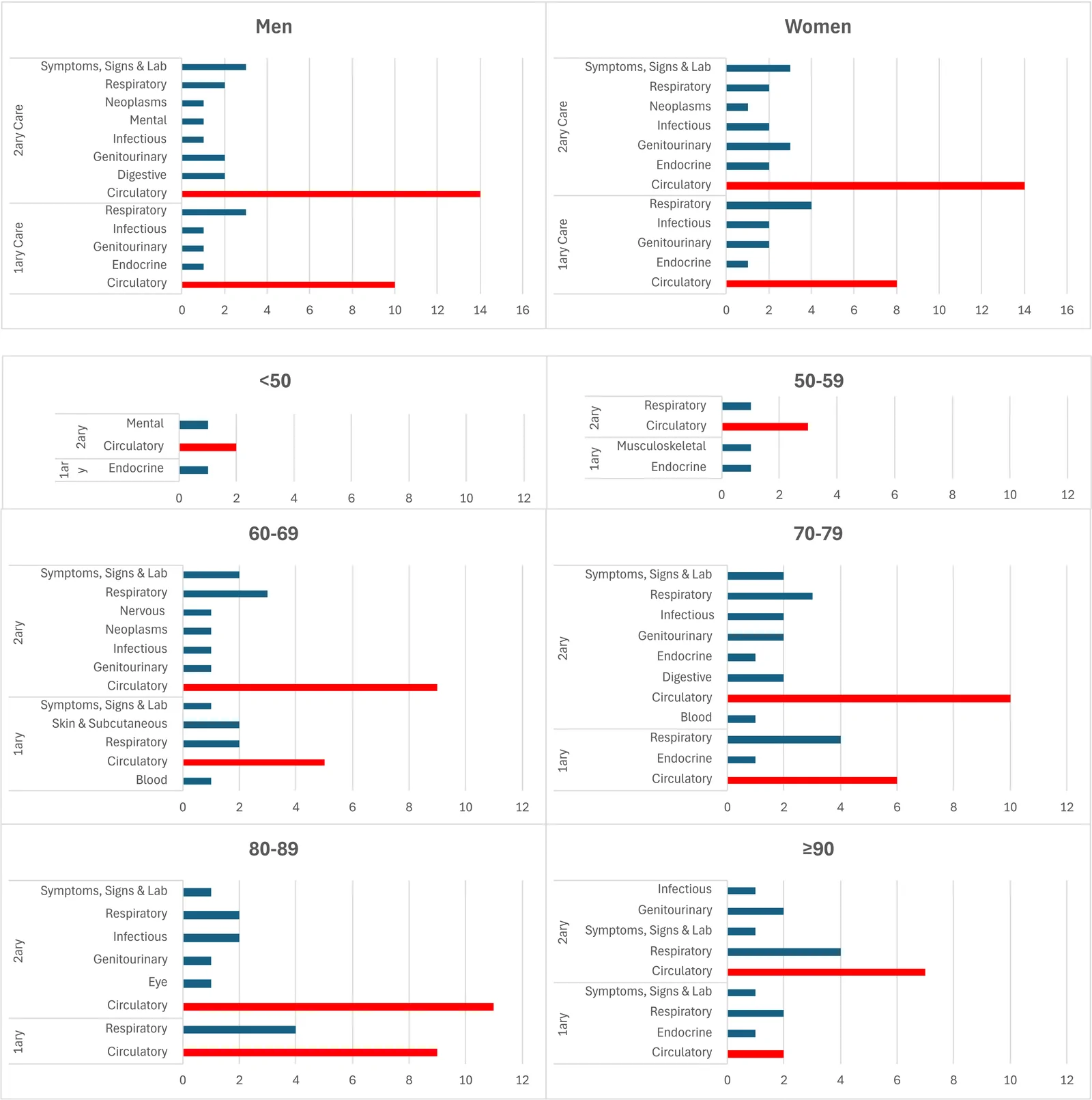
Organ Systems Defined as ICD-10 Chapters for the Associations Identified in the Primary Analyses per Predefined AF Patient Stratum Abbreviations as in Figure 1.

### Health care contacts stratified by age group

The most frequent causes for hospitalization among patients with AF differed across age strata when compared with matched control subjects (Tables 4 and 5, Figure 1B, Supplemental Figures 1 to 3).

(i) For patients with an AF diagnosis before age 50, conditions significantly more common in secondary care included “supraventricular tachycardia” (OR: 12.45; 95% CI: 7.16-23.22; *P* = 2.64 × 10^−32^), “dilated cardiomyopathy” (OR: 10.59; 95% CI: 4.65-24.82; *P* = 2.74 × 10^−8^), and “mental and behavioural disorders due to use of alcohol” (OR: 8.78; 95% CI: 3.82-20.68; *P* = 4.02 × 10^−7^), while “type 2 diabetes mellitus” was more common in primary care (OR: 4.49; 95% CI: 2.77-7.51; *P* = 2.26 × 10^−9^).
(ii) In the 50 to 59 age group, secondary care hospitalizations were more frequent for “congestive heart failure” (OR: 32.18; 95% CI: 12.13-88.80; *P* = 6.19 × 10^−12^), “supraventricular tachycardia” (OR: 23.83; 95% CI: 8.89-65.75; *P* = 4.38 × 10^−10^), “lobar pneumonia” (OR: 4.27; 95% CI: 2.58-7.41; *P* = 4.27 × 10^−8^), and dilated cardiomyopathy (OR: 14.69; 95% CI: 5.38-40.79; *P* = 1.83 × 10^−7^). In primary care, significantly higher odds were observed for “type 2 diabetes mellitus” (OR: 2.13; 95% CI: 1.73-2.74; *P* = 2.45 × 10^−11^) and gout (OR: 1.95; 95% CI: 1.55-2.52; *P* = 3.51 × 10^−8^).
(iii) For the 60 to 69 age group, patients with AF more frequently had primary care visits for 11 reasons and hospitalizations for 18 conditions. Significant associations in secondary care included “congestive heart failure” (OR: 7.81; 95% CI: 5.99-10.68; *P* = 8.70 × 10^−71^), “lobar pneumonia, unspecified” (OR: 3.51; 95% CI: 2.82-4.50; *P* = 4.11 × 10^−31^), and “left ventricular failure” (OR: 4.58; 95% CI: 3.28-6.51; *P* = 2.46 × 10^−23^). In primary care, associations included “cellulitis” (OR: 2.68; 95% CI: 2.13-3.43; *P* = 2.20 × 10^−16^), “shortness of breath” (OR: 3.80; 95% CI: 2.75-5.34; *P* = 2.20 × 10^−15^), and “acute exacerbation of chronic obstructive pulmonary disease” (OR: 3.59; 95% CI: 2.52-5.18; *P* = 2.47 × 10^−12^).
(iv) For patients aged 70 to 79, AF was associated with more frequent primary care visits for 11 conditions and secondary care admissions for 23 conditions. In primary care, notable conditions included “acute exacerbation of chronic obstructive airways disease” (OR: 4.08; 95% CI: 3.12-5.40; *P* = 5.33 × 10^−34^), “congestive heart failure” (OR: 3.49; 95% CI: 2.78-4.45; *P* = 3.23 × 10^−30^), “mitral regurgitation” (OR: 7.98; 95% CI: 4.77-13.48; *P* = 4.22 × 10^−15^), and “pulmonary embolism” (OR: 5.69; 95% CI: 3.55-9.18; *P* = 6.78 × 10^−13^). In secondary care, key associations were “lobar pneumonia, unspecified” (OR: 3.62; 95% CI: 3.22-4.26; *P* = 4.96 × 10^−89^), “aortic (valve) stenosis” (OR: 4.56; 95% CI: 3.30-6.35; *P* = 3.33 × 10^−25^), “acute renal failure, unspecified” (OR: 3.69; 95% CI: 2.82-4.87; *P* = 3.24 × 10^−25^), and “cerebral infarction due to unspecified occlusion or stenosis” (OR: 4.47; 95% CI: 3.21-6.27; *P* = 2.78 × 10^−23^).
(v) For patients with new AF diagnosed between ages 80 and 89, 13 primary care and 18 secondary care conditions were associated with more frequent health care interactions compared to control subjects. Notable secondary care findings included “congestive heart failure” (OR: 3.26; 95% CI: 3.10-3.73; *P* = 2.10 × 10^−176^), “lobar pneumonia, unspecified” (OR: 3.03; 95% CI: 2.83-3.45; *P* = 4.05 × 10^−127^), “cerebral infarction, unspecified” (OR: 2.39; 95% CI: 2.22-2.70; *P* = 9.10 × 10^−80^), “tendency to fall, not elsewhere classified” (OR: 27.50; 95% CI: 13.01-58.78; *P* = 2.59 × 10^−48^), and “atherosclerotic heart disease” (OR: 5.26; 95% CI: 4.02-6.99; *P* = 2.00 × 10^−43^). In primary care, conditions such as “pleural effusion” (OR: 5.95; 95% CI: 4.09-8.75; *P* = 6.16 × 10^−28^), “acute exacerbation of chronic obstructive airways disease” (OR: 3.91; 95% CI: 2.96-5.20; *P* = 4.15 × 10^−26^), “mitral regurgitation” (OR: 5.95; 95% CI: 3.87-9.20; *P* = 6.66 × 10^−16^), and “pulmonary embolism” (OR: 3.29; 95% CI: 2.39-4.56; *P* = 4.66 × 10^−13^) were common.
(vi) In the oldest age group (90+), 6 primary care and 15 secondary care conditions were more frequent than in control subjects, with significant secondary care associations including “congestive heart failure” (OR: 4.08; 95% CI: 3.74-5.07; *P* = 1.67 × 10^−100^), “lobar pneumonia, unspecified” (OR: 3.59; 95% CI: 3.29-4.41; *P* = 4.81 × 10^−86^), “cerebral infarction, unspecified” (OR: 2.45; 95% CI: 2.16-2.96; *P* = 3.78 × 10^−33^), “tendency to fall” (OR: 6.25; 95% CI: 3.83-10.37; *P* = 4.14 × 10^−13^), “acute myocardial infarction, unspecified” (OR: 1.99; 95% CI: 1.64-2.49; *P* = 4.16 × 10^−11^), and “urinary tract infection” (OR: 1.35; 95% CI: 1.25-1.52; *P* = 8.66 × 10^−11^).

**Table 4:** Top Reasons for Hospitalization in Atrial Fibrillation Patients, Compared to Control Subjects, Stratified by Age Groups

Age <50 y (n = 8,086)						Age 50-59 y (n = 12,749)					
Reason for Hospitalization	AF, n	Control Subjects, n	OR	95% CI	*P* Value	Reason for Hospitalization	AF, n	Control Subjects, n	OR	95% CI	*P* Value
Supraventricular tachycardia	152	12	12.5	7.2-23.2	2.6E-32	Congestive heart failure	130	4	32.2	12.1-88.8	6.2E-12
Dilated cardiomyopathy	64	6	10.6	4.7-24.8	2.7E-08	Supraventricular tachycardia	96	4	23.8	8.9-65.8	4.4E-10
Mental and behavioral disorders due to use of alcohol	53	6	8.8	3.8-20.7	4.0E-07	Dilated cardiomyopathy	59	4	14.7	5.4-40.8	1.8E-07
**60-69 (n = 31,005)**						Lobar pneumonia, unspecified	74	17	4.3	2.6-7.4	4.3E-08
Mitral (valve) insufficiency	108	3	35.6	11.5-113.8	8.9E-10	**70-79 (n = 58,992)**					
Ventricular tachycardia	73	3	24.3	7.7-77.4	5.9E-08	Cerebral infarction due to embolism of cerebral artery	73	2	36.5	9.0-148.0	5.2E-07
Dilated cardiomyopathy	51	3	17.0	5.3-54.6	1.8E-06	Cerebral infarction due to thrombosis of cerebral artery	75	7	10.7	4.9-23.3	1.9E-09
Cerebral infarction due to unspecified occlusion or stenosis	67	6	11.1	4.9-25.8	1.5E-08	Gastroenteritis and colitis of unspecified origin	73	7	10.4	4.8-22.7	3.1E-09
Supraventricular tachycardia	130	15	8.6	5.1-14.9	2.2E-15	Mitral (valve) insufficiency	125	15	8.3	4.9-14.3	8.2E-15
Precordial pain	51	6	8.5	3.7-19.9	7.1E-07	Chronic kidney disease, stage 5	52	7	7.4	3.4-16.4	6.3E-07
Congestive heart failure	411	52	7.8	6.0-10.7	8.7E-71	Tendency to fall, not elsewhere classified	59	10	5.9	3.0-11.5	2.1E-07
Sepsis, unspecified	93	15	6.2	3.6-10.7	5.2E-11	Enterocolitis due to *Clostridium difficile*	97	17	5.7	3.4-9.6	3.4E-11
Acute renal failure, unspecified	92	15	6.2	3.6-10.6	7.0E-11	Ventricular tachycardia	87	17	5.1	3.1-8.6	7.3E-10
Aortic (valve) stenosis	82	15	5.5	3.2-9.5	1.4E-09	Polyp of colon	82	17	4.8	2.9-8.1	3.5E-09
Palpitations	56	12	4.7	2.5-8.7	1.3E-0.6	Aortic (valve) stenosis	201	44	4.6	3.3-6.4	3.3E-25
Left ventricular failure	184	40	4.6	3.3-6.5	2.5E-23	Cerebral infarction due to unspecified occlusion or stenosis	188	42	4.5	3.2-6.3	2.8E-23
Lobar pneumonia, unspecified	318	90	3.5	2.8-4.5	4.1E-31	Supraventricular tachycardia	129	29	4.3	2.9-6.5	1.3E-12
Multiple myeloma	88	28	3.1	2.1-4.8	1.3E-07	Congestive heart failure	1,176	272	4.3	3.9-5.0	1.7E-135
Pneumonia, unspecified	179	68	2.6	2.0-3.5	9.8E-12	Acute renal failure, unspecified	244	66	3.7	2.8-4.9	3.3E-25
Transient cerebral ischemic attack, unspecified	145	65	2.2	1.7-3.0	7.3E-08	Lobar pneumonia, unspecified	916	250	3.6	3.2-4.3	5.0E-89
Cerebral infarction, unspecified	303	151	2.0	1.7-2.5	2.2E-12	Sick sinus syndrome	76	24	3.2	2.0-5.0	8.4E-07
Chronic obstructive pulmonary disease with acute low respiratory infection	315	157	2.0	1.7-2.4	8.1E-13	Subsequent myocardial infarction of unspecified site	121	39	3.1	2.2-4.5	7.6E-10
**80-89 (n = 67,364)**						Heart failure, unspecified	104	34	3.1	2.1-4.5	1.5E-08
Cerebral infarction due to embolism of cerebral artery	92	2	45.9	11.3-187.0	8.4E-08	Other iron deficiency anemias	95	34	2.8	1.9-4.1	2.7E-07
Tendency to fall, not elsewhere classified	193	7	27.5	13.0-58.8	2.6E-48	Other and unspecified convulsions	81	29	2.8	1.8-4.3	2.0E-06
Gastroenteritis and colitis of unspecified origin	127	16	7.9	4.7-13.4	5.6E-15	Pneumonitis due to food and vomit	114	42	2.7	1.9-3.9	3.1E-08
Cerebral infarction due to unspecified occlusion or stenosis	310	48	6.4	4.8-8.8	3.6E-48	Gastrointestinal hemorrhage, unspecified	119	44	2.7	1.9-3.8	1.6E-08
Cerebral infarction due to thrombosis of cerebral artery	68	12	5.7	3.1-10.5	3.0E-08	Pleural effusion, not elsewhere classified	248	93	2.7	2.1-3.4	6.7E-16
Atherosclerotic heart disease	317	60	5.3	4.0-7.0	2.0E-43	**≥90(n = 21,307)**					
Aortic (valve) stenosis	217	62	3.5	2.6-4.7	2.3E-21	Cerebral infarction due to unspecified occlusion or stenosis	127	9	14.0	7.2-27.9	1.5E-14
Congestive heart failure	1985	596	3.3	3.1-3.7	2.1E-176	Acute subendocardial myocardial infarction	62	9	6.9	3.4-13.9	6.1E-08
Embolism and thrombosis of arteries of lower extremities	85	28	3.0	2.0-4.7	3.4E-07	Aortic (valve) stenosis	57	9	6.3	3.1-12.8	2.6E-07
Lobar pneumonia, unspecified	1,577	513	3.0	2.8-3.5	4.0E-127	Tendency to fall, not elsewhere classified	113	18	6.3	3.8-10.4	4.1E-13
Sick sinus syndrome	83	28	3.0	1.9-4.6	6.5E-07	Congestive heart failure	877	208	4.1	3.7-5.1	1.7E-100
Enterocolitis due to *Clostridium difficile*	188	64	2.9	2.2-3.9	9.1E-14	Lobar pneumonia, unspecified	850	230	3.6	3.3-4.4	4.8E-86
Acute renal failure, unspecified	411	147	2.8	2.3-3.4	5.3E-30	Pneumonitis due to food and vomit	125	40	3.1	2.2-4.5	3.3E-10
Pleural effusion, not elsewhere classified	280	104	2.7	2.2-3.4	8.4E-20	Sepsis, unspecified	188	75	2.5	1.9-3.3	1.5E-11
Heart failure, unspecified	157	62	2.5	1.9-3.4	5.7E-10	Cerebral infarction, unspecified	540	217	2.5	2.2-3.0	3.8E-33
Subsequent myocardial infarction of unspecified site	180	74	2.4	1.9-3.2	1.2E-10	Acute renal failure, unspecified	162	66	2.4	1.9-3.3	7.0E-10
Degeneration of macula and posterior pole	132	55	2.4	1.8-3.3	4.8E-08	Pleural effusion, not elsewhere classified	96	40	2.4	1.7-3.5	3.2E-06
Cerebral infarction, unspecified	1,408	582	2.4	2.2-2.7	9.1E-80	Acute myocardial infarction, unspecified	267	133	2.0	1.6-2.5	4.2E-11
						Left ventricular failure	228	119	1.9	1.5-2.4	7.7E-09
						Pneumonia, unspecified	589	310	1.9	1.7-2.2	3.5E-21
						Urinary tract infection, site not specified	995	730	1.4	1.3-1.5	8.7E-11

All results pass the threshold for multiple adjusted comparisons of *P* < 0.0000038 (ie, *P* < 3.8E-06).

AF = atrial fibrillation.

**Table 5:** Top Reasons for Primary Care Consultations in Atrial Fibrillation Patients, Compared to Control Subjects, Stratified by Age Groups

Age <50 y (n = 8,086)						Age 50-59 y (n = 12,749)					
Reason for Primary Care Visit	AF, n	Control Subjects, n	OR	95% CI	*P* Value	Reason for Primary Care Visit	AF, n	Control Subjects, n	OR	95% CI	*P* Value
Type 2 diabetes mellitus	86	19	4.5	2.8-7.5	2.3E-09	Type 2 diabetes mellitus	235	109	2.1	1.7-2.7	2.5E-11
**60-69 (n = 31,005)**						Gout	196	100	2.0	1.6-2.5	3.6E-08
Mitral regurgitation	86	4	21.4	7.9-58.8	1.9E-09	**70-79 (n = 58,992)**					
Congestive cardiac failure	76	7	10.8	5.02-23.6	1.5E-09	Mitral regurgitation	128	16	8.0	4.8-13.5	4.2E-15
Congestive heart failure	119	12	9.9	5.5-18.0	3.4E-14	Thyrotoxicosis	59	10	5.9	3.0-11.5	2.1E-07
Heart failure	69	7	9.8	4.5-21.5	7.8E-09	Pulmonary embolism	114	20	5.7	3.6-9.2	6.8E-13
Pneumonia due to unspecified organism	92	17	5.4	3.2-9.1	1.5E-10	Acute non-ST-segment elevation myocardial infarction	102	20	5.1	3.2-8.3	2.6E-11
Anemia unspecified	70	17	4.1	2.4-7.0	1.6E-07	Heart failure	157	33	4.8	3.3-6.9	4.4E-16
Left ventricular failure	104	27	3.8	2.5-5.9	4.1E-10	Acute exacerbation of chronic obstructive airways disease	262	64	4.1	3.1-5.4	5.3E-34
Shortness of breath	168	44	3.8	2.8-5.3	2.2E-15	Pleural effusion NOS	97	24	4.0	2.6-6.3	8.8E-10
Acute exacerbation of chronic obstructive airways disease	137	38	3.6	2.5-5.2	2.5E-15	Congestive cardiac failure	173	49	3.5	2.6-4.9	6.0E-15
Cellulitis NOS	253	94	2.7	2.1-3.4	2.2E-16	Congestive heart failure	312	89	3.5	2.8-4.5	3.2E-30
Leg ulcer NOS	109	44	2.5	1.8-3.5	3.7E-07	Pneumonia due to unspecified organism	160	54	3.0	2.2-4.0	4.9E-12
**80-89 (n = 67,364)**						Lower respiratory tract infection	90	31	2.9	1.9-4.4	3.0E-07
Acute non-ST-segment elevation myocardial infarction	196	22	8.9	5.8-13.9	4.0E-36	**≥90 (n = 21,307)**					
Acute coronary syndrome	74	9	8.2	4.1-16.4	2.4E-09	Acute non-ST-segment elevation myocardial infarction	75	3	24.9	7.9-79.6	4.5E-08
Pleural effusion NOS	185	31	6.0	4.1-8.8	6.2E-28	Pneumonia due to unspecified organism	217	59	3.7	2.8-4.9	1.5E-22
Mitral regurgitation	143	24	6.0	3.9-9.2	6.7E-16	Shortness of breath	131	40	3.3	2.3-4.7	4.7E-11
Acute exacerbation of chronic obstructive airways disease	239	61	3.9	3.0-5.2	4.2E-26	Lower respiratory tract infection	87	27	3.2	2.1-5.0	1.0E-07
Cerebral arterial occlusion	72	20	3.8	2.3-6.2	1.5E-07	Type 2 diabetes mellitus	112	38	2.9	2.1-4.3	8.0E-09
CVA—cerebral artery occlusion	145	39	3.7	2.6-5.3	3.2E-13	Congestive heart failure	246	132	1.9	1.5-2.3	6.8E-09
Heart failure	293	88	3.3	2.6-4.2	6.4E-27						
Pulmonary embolism	158	48	3.3	2.4-4.6	4.7E-13						
Cerebral infarction NOS	116	37	3.1	2.2-4.6	1.4E-09						
Lower respiratory tract infection	198	72	2.7	2.1-3.6	1.9E-13						
Pneumonia due to unspecified organism	437	160	2.7	2.3-3.3	8.5E-31						
Congestive cardiac failure	412	160	2.6	2.2-3.1	1.1E-26						

All results pass the threshold for multiple adjusted comparisons of *P* < 0.0000005 (ie, *P* < 5E-07).

Abbreviations as in Table 3.

There was a predominance of the circulatory system as the most commonly associated organ system for all age groups, with the number of associations meeting the multiple-adjusted significance threshold more frequently in patients with AF ranging from 2 to 20. The second most commonly involved organ system was the respiratory system, with the number of associations ranging from 5 to 7 in all groups between 60 and over 90 (Figure 2).

### Secondary analyses

An analysis of conditions meeting the discovery *P* value threshold (*P* < 0.0005) revealed additional associations compared to matched control subjects (Supplemental Appendix). For women with AF, 16 additional associations were found in primary care and 16 in secondary care, when compared to age-matched control subjects. These included primary pulmonary hypertension, intracerebral hemorrhage, subarachnoid hemorrhage, and anticoagulant-related bleeding, hypokalemia, malignant bronchial neoplasm, fragility fracture, ascites, and calculus of bile duct with cholangitis. For men, 16 additional primary care and 22 secondary care conditions were significantly associated with AF, including, but not limited to, cardiac arrest, ventricular fibrillation and flutter, ischemia of the legs, vascular disorders of the intestine, multiple myeloma, seizures, neuropathic pain, and aspiration pneumonitis.

For patients with AF aged <50 years, several additional conditions were identified when compared to matched control subjects, including gout, alcohol-dependence syndrome, thyrotoxicosis, mitral regurgitation, atherosclerotic/ischemic heart disease, chest pain, and palpitations. Among patients aged 50 to 59, 4 additional causes for hospitalization and 8 reasons for primary care visits were found, which included cerebral infarction, left ventricular failure, ischemic heart disease, and cellulitis. The 60 to 69 age group showed 11 additional reasons for primary care visits and 9 for hospitalization, with examples including lung cancer, pleural effusion, thyrotoxicosis, and cerebrovascular accident. In their seventies, patients with AF had 10 additional conditions in primary care and 8 conditions in secondary care that were more common than in control subjects, including endocarditis, sepsis, hyperkalemia, lung cancer, acute coronary syndrome, and primary pulmonary hypertension. Analyses of patients in their eighties revealed 10 more conditions in primary care and 8 more secondary care conditions that were more frequent in patients with AF, including polyps of colon, secondary malignant neoplasms of the pleura, gout, respiratory failure, acute renal failure, fragility fracture, and traumatic subdural hemorrhage. Finally, patients with AF aged ≥90 experienced eight additional conditions in primary care and 7 in secondary care more frequently than their matched control subjects. These conditions included “subdural hemorrhage (acute) (nontraumatic)”, “embolism and thrombosis of arteries of lower extremities”, “pulmonary embolism”, “sick sinus syndrome”, “myelodysplastic syndrome, unspecified”, and “hypo-osmolality and hyponatremia”.

The secondary analysis using a positive control group of 150,182 patients with ischemic heart disease showed a similar pattern of associations (Supplemental Tables 2 and 3, Supplemental Appendix). Albeit ischemic heart disease was no longer one of the top 100 associations, other associations with heart failure, cardiomyopathies, heart valve disease, sepsis and respiratory infections, neoplastic disorders, obesity, and alcohol-abused related disorders remained more frequent in the AF group (Supplemental Tables 2 and 3). Most of the identified significant associations were with circulatory system conditions (Supplemental Figure 4).

### Literature review and evaluation of causality

After grouping the identified associations from our primary analyses, we found 51 phenotypes that were more common in patients with AF than in age- and sex-matched control subjects. On September 15, 2025, we conducted 51 searches to identify previous reports on the association of AF with the phenotypes. Of these, 28 associations had been previously reported in observational studies,^17^^,^^21^, ^22^, ^23^, ^24^, ^25^, ^26^, ^27^, ^28^, ^29^, ^30^, ^31^, ^32^, ^33^, ^34^, ^35^, ^36^, ^37^, ^38^, ^39^, ^40^, ^41^, ^42^, ^43^, ^44^, ^45^ while the remaining associations appear to be potentially novel.

On the same date, we conducted 51 searches for MR studies evaluating AF as the exposure in relation to the phenotypes identified earlier. Of these, MR analyses suggested that AF has a causal effect on 19 phenotypes and no causal effect on 10 others.^46^, ^47^, ^48^, ^49^, ^50^, ^51^, ^52^, ^53^, ^54^, ^55^, ^56^, ^57^, ^58^, ^59^, ^60^, ^61^ A detailed description of the identified associations and MR studies is presented in Table 6.

**Table 6:** Phenotypes More Frequently Observed in the Different Sex- and Age-Related Strata in the Primary Analyses and Literature Review

Phenotype	Known vs Novel Association	Causality/MR	Men	Women	Age <50 y	Age 50-59 y	Age 60-69 y	Age 70-79 y	Age 80-89 y	Age ≥90 y
Abnormal findings of blood chemistry	Known^21^	✓^46^		✓						
Acute kidney failure	Known^22^	✓^47^	✓	✓			✓	✓	✓	✓
Acute myeloblastic leukemia	Novel		✓							
Age-related macular degeneration	Known^23^	X^48^							✓	
Anemia (unspecified/iron deficiency)	Novel	✓^49^					✓	✓		
Aortic valve stenosis	Novel	X^50^	✓	✓			✓	✓	✓	✓
Aortic valve regurgitation	Novel		✓							
Aspiration pneumonia/Pneumonitis	Novel									✓
Barret’s esophagus	Novel		✓							
Cellulitis	Novel						✓			
Chest pain/Precordial pain	Known^24^		✓	✓			✓			
Chronic obstructive pulmonary disease (with exacerbation)	Novel	X^51^	✓	✓			✓	✓	✓	
Convulsions	Known^25^ (epilepsy)	✓^52^								
Diabetes mellitus (type 2)	Novel	X^53^			✓	✓				✓
Dilated cardiomyopathy	Novel	✓^54^	✓		✓	✓	✓			
Disorders of both mitral and aortic valves	Novel	✓^55^	✓							
Disorders of both mitral and tricuspid valves	Novel	✓^55^		✓						
Diffuse large B-cell lymphoma	Novel	X^56^		✓						
End-stage renal disease	Known^22^	✓^47^	✓	✓				✓		
Enterocolitis due to *Clostridium difficile*	Novel			✓				✓	✓	
Falls	Known^26^		✓	✓				✓	✓	✓
Gastroenteritis and colitis of unspecified origin	Novel	X^48^	✓	✓				✓	✓	
Gastrointestinal hemorrhage	Novel							✓		
Gout	Novel	X^57^				✓				
Leg ulcer	Known^27^						✓			
Left ventricular failure	Known^28^	✓^48^					✓			✓
Heart failure (congestive)	Known^29^	✓^53^	✓	✓			✓	✓	✓	✓
Hyperkalemia	Novel			✓						
Infective endocarditis	Novel		✓	✓						
Ischemic stroke—embolic or atherosclerotic	Known^30^	✓^53^/^48^	✓	✓			✓	✓	✓	✓
Mental and behavioral disorders due to the use of alcohol	Novel		✓		✓					
Mitral valve regurgitation	Known^31^	✓^58^	✓	✓			✓	✓	✓	
Myocardial infarction/Coronary artery disease	Known^32^^,^^33^	✓^59^ (MI only)	✓	✓				✓	✓	✓
Multiple myeloma	Known^34^						✓			
Palpitations	Known^24^		✓				✓			
Peripheral artery disease	Known^35^	✓^60^								
Pleural effusion	Novel	✓^48^	✓	✓				✓	✓	✓
Pneumonia/Lower respiratory tract infection	Known^36^	X^48^	✓	✓		✓	✓	✓	✓	✓
Polyp of colon	Known^37^	X^48^	✓					✓		
Primary pulmonary hypertension	Known^38^		✓							
Pulmonary embolism	Known^39^	X^48^	✓	✓				✓	✓	
Respiratory failure	Known^40^			✓						
Sepsis/Septicemia	Known^41^		✓	✓			✓			✓
Shortness of breath	Known^24^						✓			✓
Sick sinus syndrome	Known^42^	✓^61^	✓	✓				✓	✓	
Supraventricular tachycardia	a	✓^48^	✓	✓	✓	✓	✓	✓		
Systemic embolism	Known^43^	✓^53^		✓					✓	
Thyrotoxicosis	Known^44^		✓	✓				✓		
Transient ischemic attack	Known^45^	✓^60^					✓			
Urinary tract infection	Known^4^									✓
Ventricular tachycardia/Cardiac arrest/Sudden cardiac death	Known^17^		✓	✓			✓	✓		

However, mechanism-wise AF does not meet the typical supraventricular tachycardia criteria, which usually assume these are regular, re-entrant tachycardias.^62^ Interestingly, Katsiritis et al^63^ were able to induce supraventricular tachycardia in 7.6% of patients referred for AF ablation, which is above the prevalence of supraventricular tachycardia in the normal population.^64^ However, no case control study has yet assessed this.

AF = atrial fibrillation; MI = myocardial infarction; MR = Mendelian randomization.

a In a broad sense, AF can be considered a supraventricular arrhythmia as it originates above the ventricles.

## Discussion

In this study, we used nationwide data to characterize health care interactions among men and women recently diagnosed with AF, as well as across different age groups. We found that the health care encounters most frequent in the first 5 years following an AF diagnosis varied by sex and age and extended beyond cardiovascular conditions (Central Illustration). These findings highlight the heterogeneity of AF and may inform precision medicine approaches, as comorbidity profiles and AF-related complications differ across patient subgroups. Importantly, we also examined interactions within both primary and secondary care. This distinction matters because patients engage with these settings for different reasons, and failing to consider the full spectrum of health care contacts risks providing an incomplete picture of AF-related trajectories.

**Central Illustration:**
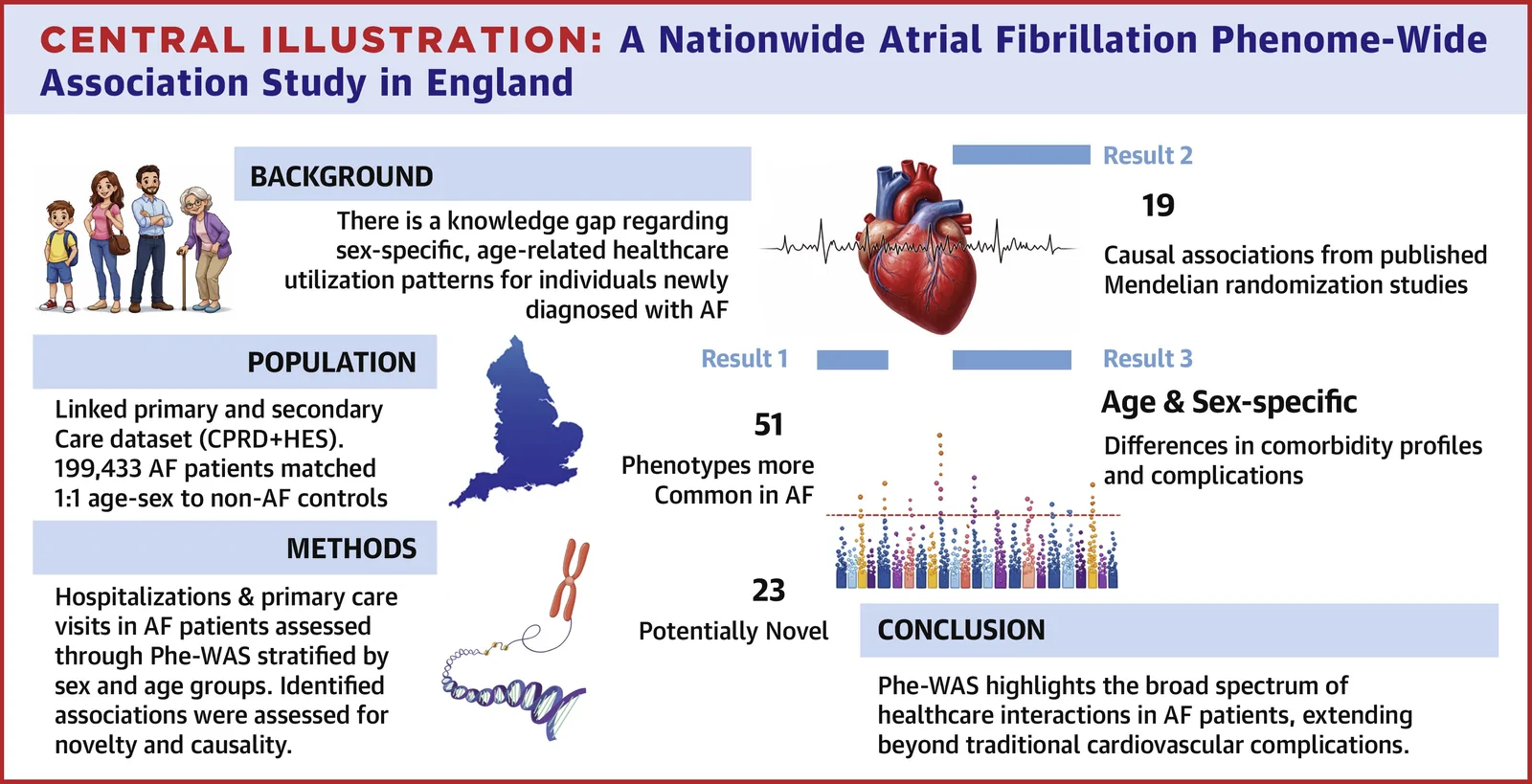
A Nationwide Atrial Fibrillation Phenome-Wide Association Study in England

Our findings confirm established knowledge on AF-related complications while also highlighting potential novel associations. Cardiovascular conditions such as congestive heart failure, stroke, and atherosclerotic heart disease were among those with the strongest associations with AF, but notable noncardiovascular conditions, such as pneumonia, showed similarly strong links. We also identified additional cardiovascular conditions—including pulmonary embolism, ventricular arrhythmias and sudden cardiac arrest, mitral regurgitation, and aortic stenosis—that occurred more frequently in the AF population and may warrant a targeted assessment and risk stratification in newly diagnosed patients. Although registry data suggest that some of these conditions (eg, aortic stenosis) are common in patients with AF, they had not previously been demonstrated to occur more often in AF than in age- and sex-matched control subjects.

Women with AF appeared to experience significantly more bleeding complications (eg, intracerebral and subarachnoid hemorrhage), fragility fractures, *Clostridium difficile* enterocolitis, cholangitis secondary to bile duct calculi, respiratory failure, and electrolyte disturbances such as hypokalemia and hyperkalemia compared with sex-matched control subjects. By contrast, men with AF had higher rates of mental and behavioral disorders related to alcohol use, ventricular fibrillation, cardiac arrest with successful resuscitation, cardiomyopathy, vascular disorders of the intestine, Barrett’s esophagus, colon polyps, bowel obstruction, osteomyelitis, and neuropathic pain. Sex-specific differences were also observed in neoplastic associations: Men with AF had significantly higher rates of acute myeloid leukemia and multiple myeloma, whereas women with AF had more malignant bronchial neoplasms and diffuse large B-cell lymphoma.

Mental and behavioral disorders related to alcohol use, gout, and dilated cardiomyopathy were distinguishing conditions more common in younger age groups (<50, 50-59, and <70 years, respectively). Pneumonia and lower respiratory tract infections were significantly more frequent across all age groups above 50. Conditions such as heart failure, ischemic stroke, acute kidney failure, enterocolitis, aortic valve stenosis, and exacerbations of chronic obstructive pulmonary disease became significantly more common after the age of 60 or 70, while urinary tract infections were particularly increased in those over 90. Supraventricular tachycardia was more common in all age groups below 80.

A Danish nationwide electronic health record study evaluated the impact of age at the time of diagnosis on the incidence of cardiovascular events (cardiomyopathy, heart failure, and stroke) and all-cause mortality.^5^ Similarly, a UK Biobank analysis examined dementia risk in patients with AF according to age at diagnosis.^6^ Prior work from the Global RE-LY AF Registry assessed sex differences in treatment and outcomes, focusing mainly on stroke, heart failure admissions, and death following emergency department visits.^8^ Likewise, the PREFER AF registry investigated sex differences in patients with AF, with emphasis on stroke and systemic embolism, acute coronary syndrome, heart failure, and bleeding.^9^ Our analysis builds on these cardiovascular-focused studies by providing a broader perspective on AF, capturing its overall complexity and the full spectrum of health care system interactions.

At a time when comprehensive management of AF is increasingly guided by structured approaches—such as the HEAD2TOES checklist for systematic comorbidity assessment^65^ and evidence-based interventions such as the Atrial Fibrillation Better Care Pathway,^66^, ^67^, ^68^ which has been shown to improve patient outcomes—it is crucial to gain a more complete understanding of the full spectrum of complications affecting the AF population, with particular attention to sex- and age-specific risks. Our study contributes to this agenda by providing evidence on novel and less well-recognized associations, including pneumonia, sepsis, and certain cancers. These findings underscore the importance of preventive strategies (eg, vaccination and immunization), earlier detection and screening, and timely initiation of treatment among identified high-risk groups.

Despite the strong observational associations identified in our Phe-WAS, our literature review revealed that MR did not always support a causal role for AF. A clear example of this discrepancy is pulmonary embolism. While observational studies, such as Hald et al,^39^ suggest that AF is a cause-specific risk factor for pulmonary embolism, and our own findings demonstrate a higher incidence of pulmonary embolism in individuals with AF, genetic causality remains unconfirmed by MR investigations.^48^ However, a negative MR finding does not definitively rule out causality. Methodological limitations, such as a lack of statistical power or weak genetic instruments for AF, can often account for the failure to detect a causal relationship.^69^ Importantly, even if some associations lack causality, they may still be relevant (eg, patients with AF should be considered a population with higher pretest probability for PE when presenting with compatible symptoms).

Our study spans throughout nearly 2 decades, with important changes occurring in profile and treatment of patients with AF. Anticoagulant use increased throughout the study period. Direct oral anticoagulants (DOACs) were introduced in the United Kingdom between 2012 and 2015. As previously shown by Wu et al in an analysis of over 3,000 UK general practices, the annual increase in UK anticoagulant use occurred primarily from 2014 onwards (+5% per year, compared to +1%-2% in 2012-2013). By 2018, three-quarters of patients with a CHA_2_DS_2_-VASc score ≥2 were treated with oral anticoagulants.^70^ In the same year, DOACs accounted for 60% of all anticoagulant prescriptions in the AF population, confirming the shift from vitamin K antagonists to DOACs. Consistent with our cohort's findings, the rate of ischemic stroke among UK patients with AF increased until 2012/2013—alongside a rising number of comorbidities—before declining afterwards as oral anticoagulant uptake increased.^71^ Trends for pulmonary embolism in our cohort mirrored those described for the wider UK population by Zhong et al^72^, showing steady levels with a slight upward long-term trend between 2008 and 2015.

### Study strengths and limitations

The strengths of our work include the discovery power afforded by a large, nationwide data set, which enabled the identification of numerous associations despite stringent significance thresholds, and the use of MR to help validate associations and assess causality. Nonetheless, several limitations warrant consideration. First, the data set is limited to the English population and may not be generalizable to other countries or regions. Second, the main analysis did not match for comorbidities or health care-seeking behavior. Consequently, the control group may have been healthier overall than the AF cases. This difference introduces a potential detection bias that could overestimate the observed associations. To address this, we conducted a secondary analysis using positive control subjects with coronary artery disease. This secondary analysis confirmed most of the primary findings. Third, non-White ethnic groups are underrepresented. This is relevant given the known ethnic differences in clinical epidemiology and complications associated with AF.^73^^,^^74^ Third, as with all observational analyses, residual confounding cannot be excluded; we sought to mitigate this by validating our findings against prior observational and MR evidence. Finally, anticoagulant use for AF in England was relatively low during the study period (1998-2016), which may have influenced the observed strength of associations with thromboembolic outcomes such as stroke and pulmonary embolism. These findings therefore primarily reflect the natural history of largely untreated AF and its complications.

## Conclusions

Our Phe-WAS confirmed well-established AF-related complications while also identifying potential novel associations and demonstrating that the broad spectrum of health care interactions in patients with AF extends beyond traditional cardiovascular risk factors and complications. These findings enhance our understanding of health care utilization in the AF population across both primary and secondary care. Importantly, the observed age- and sex-specific differences identified in our study underscore opportunities to further tailor AF management and therapy.

Perspectives **COMPETENCY IN MEDICAL KNOWLEDGE:** Atrial fibrillation (AF) manifests with significant sex- and age-specific clinical heterogeneity that extends far beyond traditional cardiovascular complications. Within 5 years of diagnosis, patients with AF experience a marked increase in both cardiovascular (congestive heart failure, valvular heart disease, pulmonary embolism) and noncardiovascular health care interactions (pneumonia, falls, acute kidney injury, and specific malignancies). Women present higher postdiagnosis risks for major bleeding, fragility fractures, and respiratory failure, whereas men demonstrate higher risks of ventricular arrhythmias, dilated cardiomyopathy, and alcohol-related disorders. Younger patients with AF (<50 years) frequently present with alcohol dependency, type 2 diabetes, and supraventricular tachycardia, whereas elderly patients (≥80 years) predominantly interact with the health care system for falls, pneumonia, stroke, and renal failure.

**TRANSLATIONAL OUTLOOK:** While Phe-WAS reveal a broad spectrum of post-AF diagnoses, Mendelian randomization analyses demonstrate that not all observational associations, such as pulmonary embolism, represent direct causal relationships. Future translational and clinical studies should focus on validating these novel noncardiovascular associations to distinguish true downstream sequelae from shared systemic risk pathways. Clinically, these findings support shifting from a purely cardiovascular management model to structured, multidisciplinary care plans (such as the ABC pathway and HEAD2TOES checklist) tailored to an individual’s age- and sex-specific risk profile, incorporating targeted screening, immunization strategies, and proactive fall prevention.

### Data availability statement

Data may be obtained from a third party and are not publicly available. Data used in this study were accessed through NHS Digital that is subject to protocol approval and cannot directly be shared. This study is based in part on data from the Clinical Practice Research Datalink obtained under license from the UK Medicines and Healthcare products Regulatory Agency. The data are provided by patients and collected by the NHS as part of their care and support. The interpretation and conclusions in this study are those of the author/s alone. The OPCS Copyright© 2020 was re-used with the permission of The Health & Social Care Information Centre. All rights reserved. Classification of interventions and procedures, codes, terms, and text is Crown copyright (2016) published by the Health and Social Care Information Centre, also known as NHS Digital and licensed under the Open Government License available at www.nationalarchives.gov.uk/doc/open-government-licence/open-government-licence.htm.

## Funding support and author disclosures

Dr Chung is supported by the National Institute for Health and Care Research, England, (10.13039/100006662NIHR) grants NIHR131227, NIHR129463, UKRI/ERC/HORIZON 10103153 Aristoteles, and UKRI/HORIZON EP/Z000211/1 AF-MED. Mr Yoshimura is supported by UKRI/ERC/HORIZON 10103153 Aristoteles. Dr Lip is a NIHR Senior Investigator and co-PI of the AFFIRMO project on multimorbidity in AF (899871), TARGET project on digital twins for personalized management of atrial fibrillation and stroke (101136244), and ARISTOTELES project on artificial intelligence for management of chronic long-term conditions (101080189), which are all funded by the EU's Horizon Europe Research and Innovation programme. Dr Providencia is supported by the UCL British Heart Foundation (BHF) Research Accelerator AA/18/6/34223, 10.13039/100006662NIHR grant NIHR129463, UKRI/ERC/HORIZON 10103153 Aristoteles, and UKRI/HORIZON EP/Z000211/1 AF-MED. Dr Schmidt is supported by the BHF grant PG/18/5033837, the UCL BHF Research Accelerator AA/18/6/34223, UKRI/ERC/HORIZON 10103153 Aristoteles, and UKRI/HORIZON EP/Z000211/1 AF-MED.
