# Supplementary material for: Decoding Atrial Fibrillation Heterogeneity: A Nationwide Phenome-Wide Association Study in England
Source: JACC Adv. 2026 Oct 22;5(11):103352. doi: 10.1016/j.jacadv.2026.103352 (PMC13718782; doi:10.1016/j.jacadv.2026.103352)
Supplement: Supplementary file 1 [file mmc1.pdf]

# Supplemental Appendix

**Figure S-1.** Trends in utilization of anticoagulants and rate of ischemic stroke, venous thromboembolism and major bleeding in the AF patient cohort.......... 2

**Figure S-2.** Manhattan plots showing medcodes more frequently identified in AF patients.......................................................................................... 3

**Figure S-3.** Manhattan plots showing ICD-10 codes more frequently identified AF patients ......................................................................... 7

**Figure S-4.** Organ systems defined as ICD-10 chapters for the associations identified in the secondary analyses (AF without ischemic heart disease vs Ischemic heart disease without AF at baseline) per pre-defined AF patient stratum......................................................................................... 11

**Table S-1.** Utilized Electronic Health Record (EHR) codes.........................................12

**Table S-2.** Top reasons for primary care consultations in AF patients without ischemic heart (IHD) disease at baseline, compared to controls with IHD......................................................................................... 13

**Table S-3.** Top reasons for hospitalisation in AF patients without IHD at baseline, compared to controls with IHD.......................................................................14

**Figure S-1.** Trends in utilization of anticoagulants and rate of ischemic stroke, venous thromboembolism and major bleeding in the AF patient cohort.

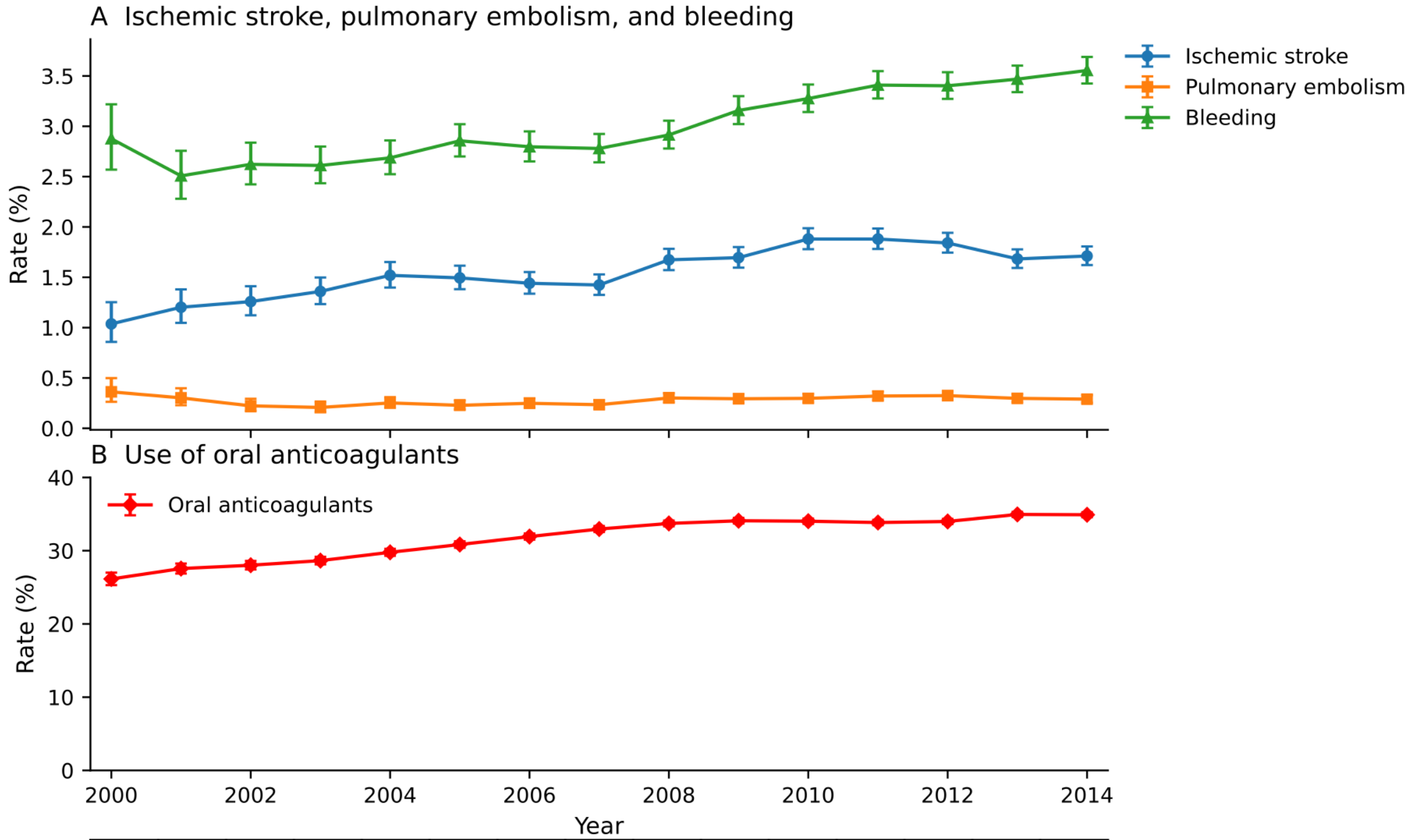


| | 2000 | 2001 | 2002 | 2003 | 2004 | 2005 | 2006 | 2007 | 2008 | 2009 | 2010 | 2011 | 2012 | 2013 | 2014 |
|---|---|---|---|---|---|---|---|---|---|---|---|---|---|---|---|
| No. at risk | 10,222 | 16,552 | 22,881 | 29,497 | 35,735 | 41,631 | 47,130 | 52,133 | 56,827 | 61,064 | 65,303 | 68,664 | 71,864 | 74,352 | 75,732 |

**Figure S-2.** Manhattan plots showing medcodes more frequently identified AF patients

## A. Men

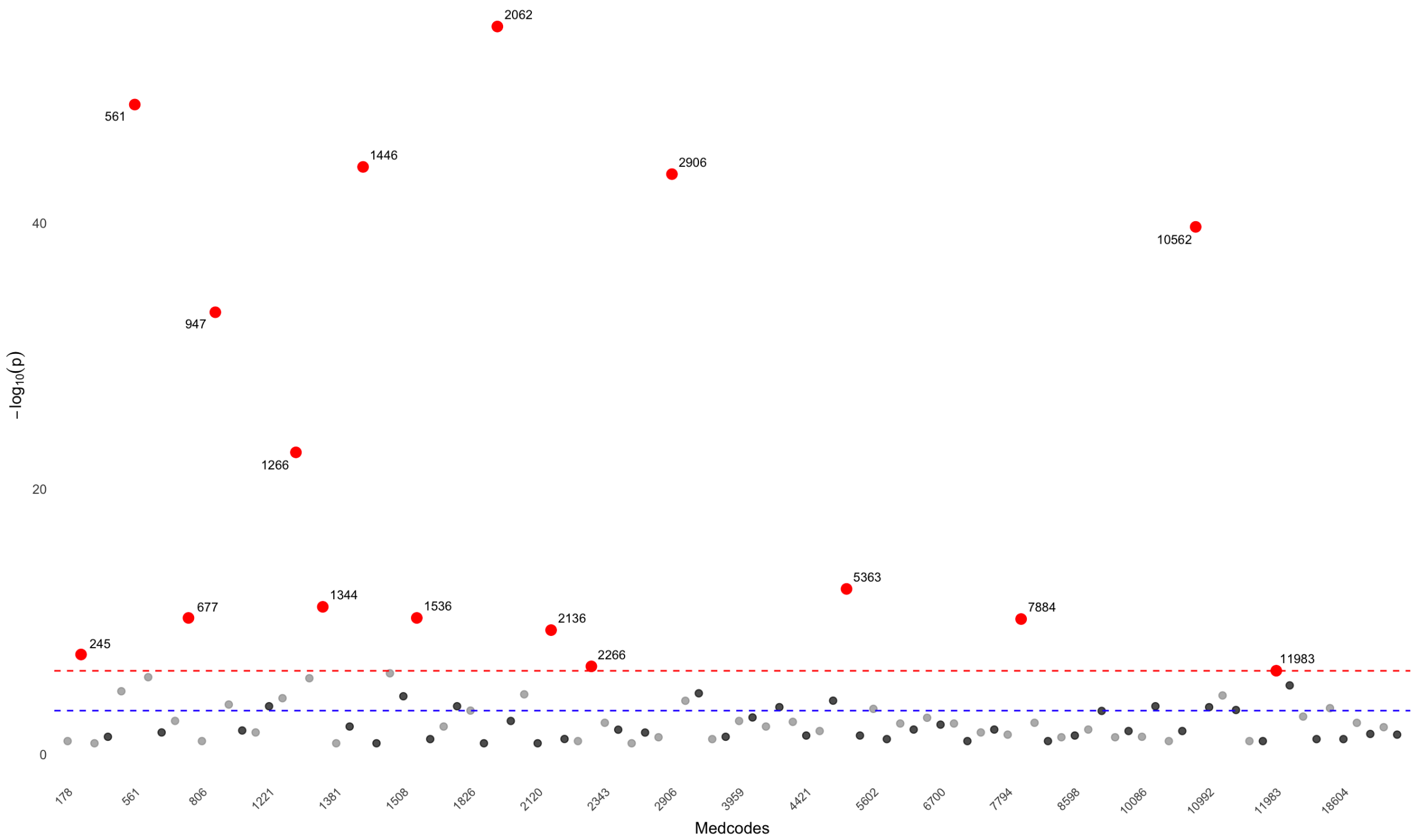


## B. Women

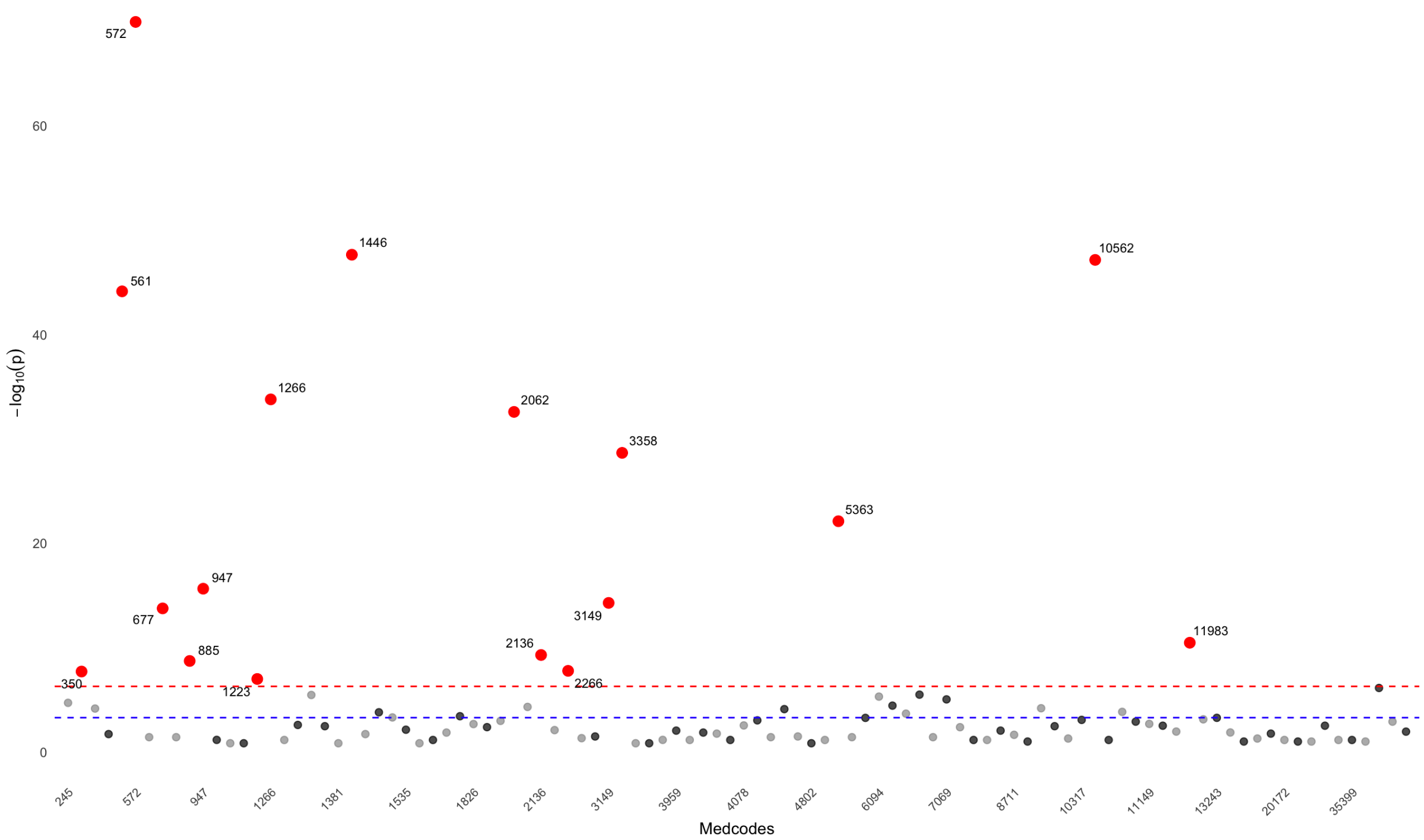

## C. <50 years

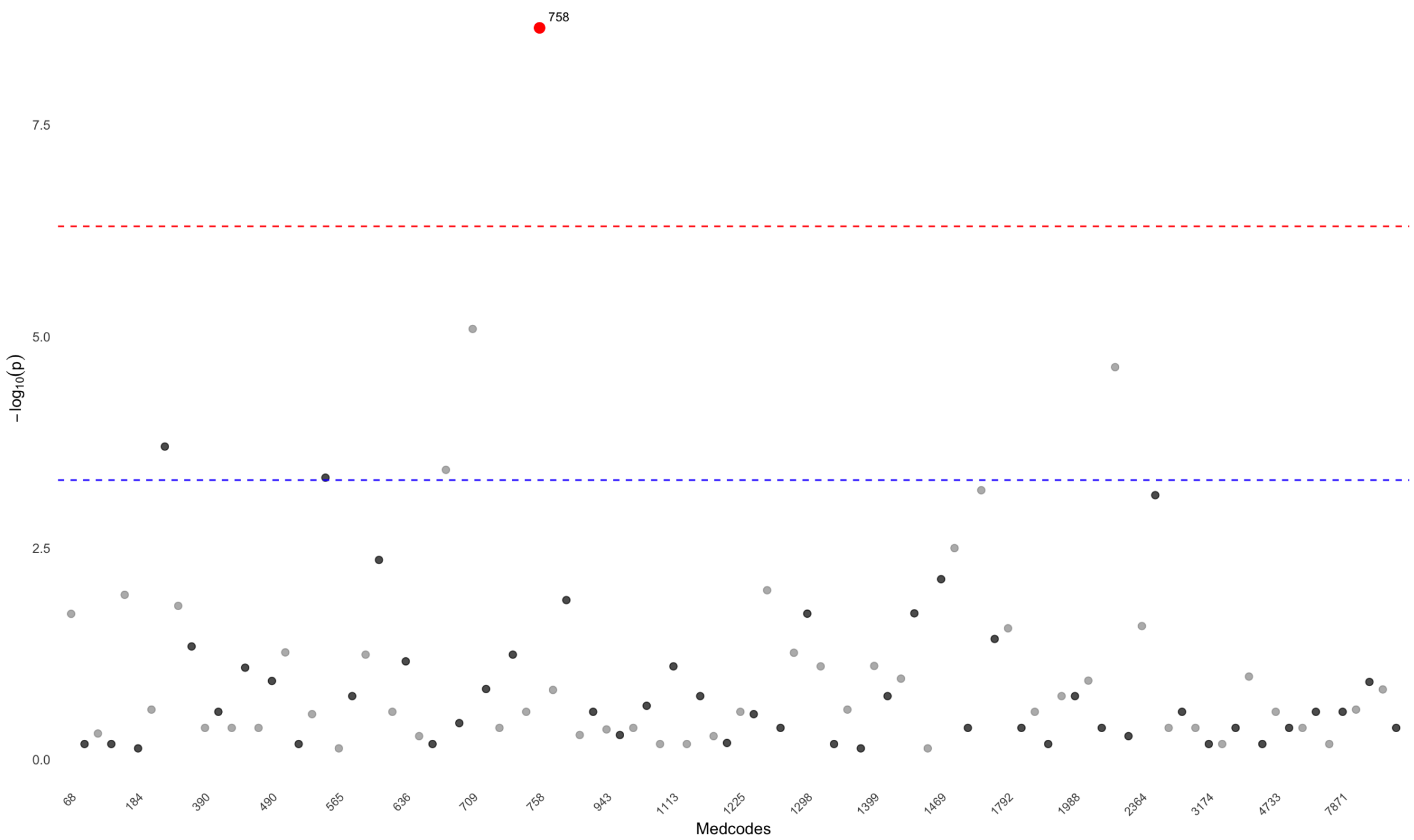


## D. 50 to 59 years

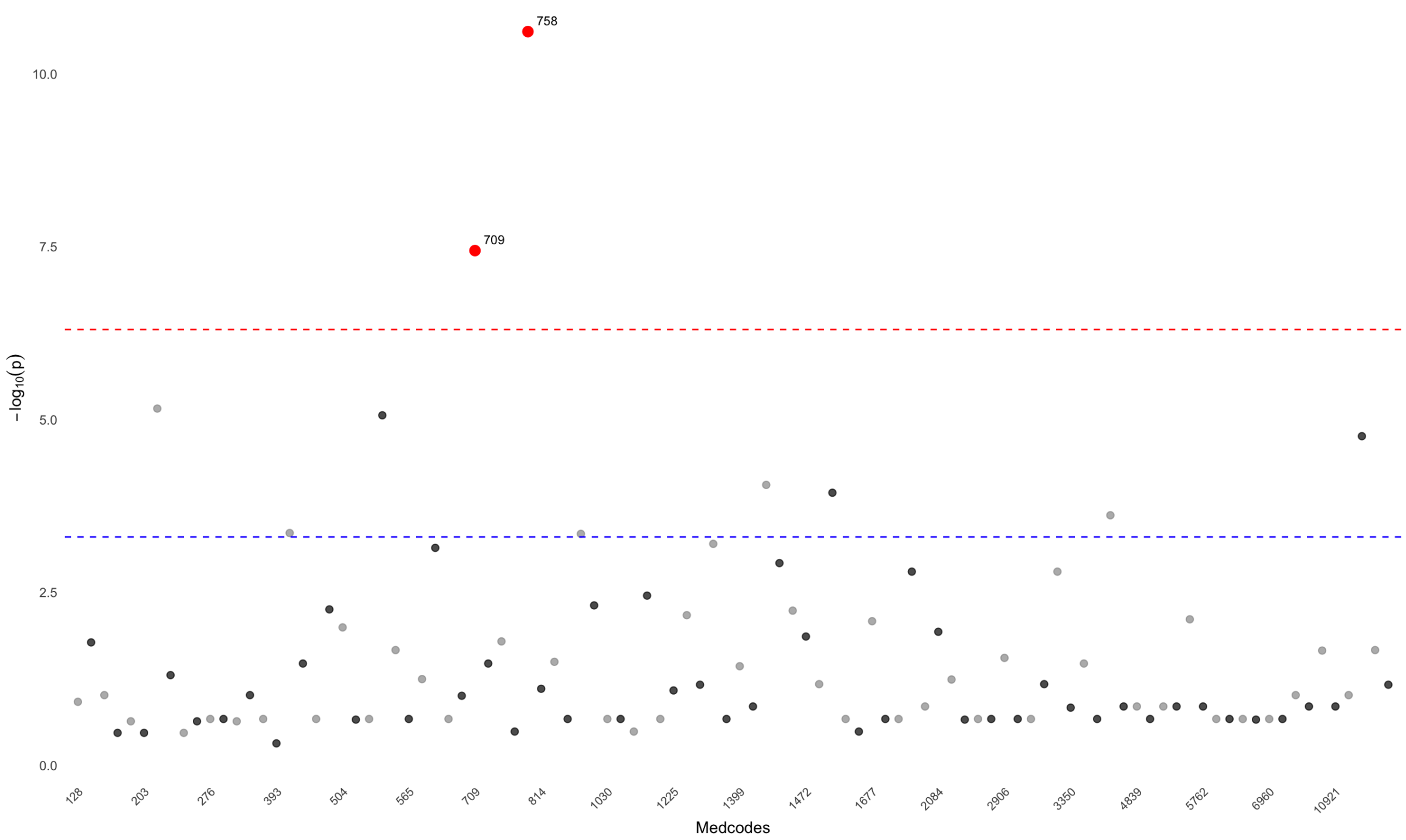

## E. 60 to 69 years

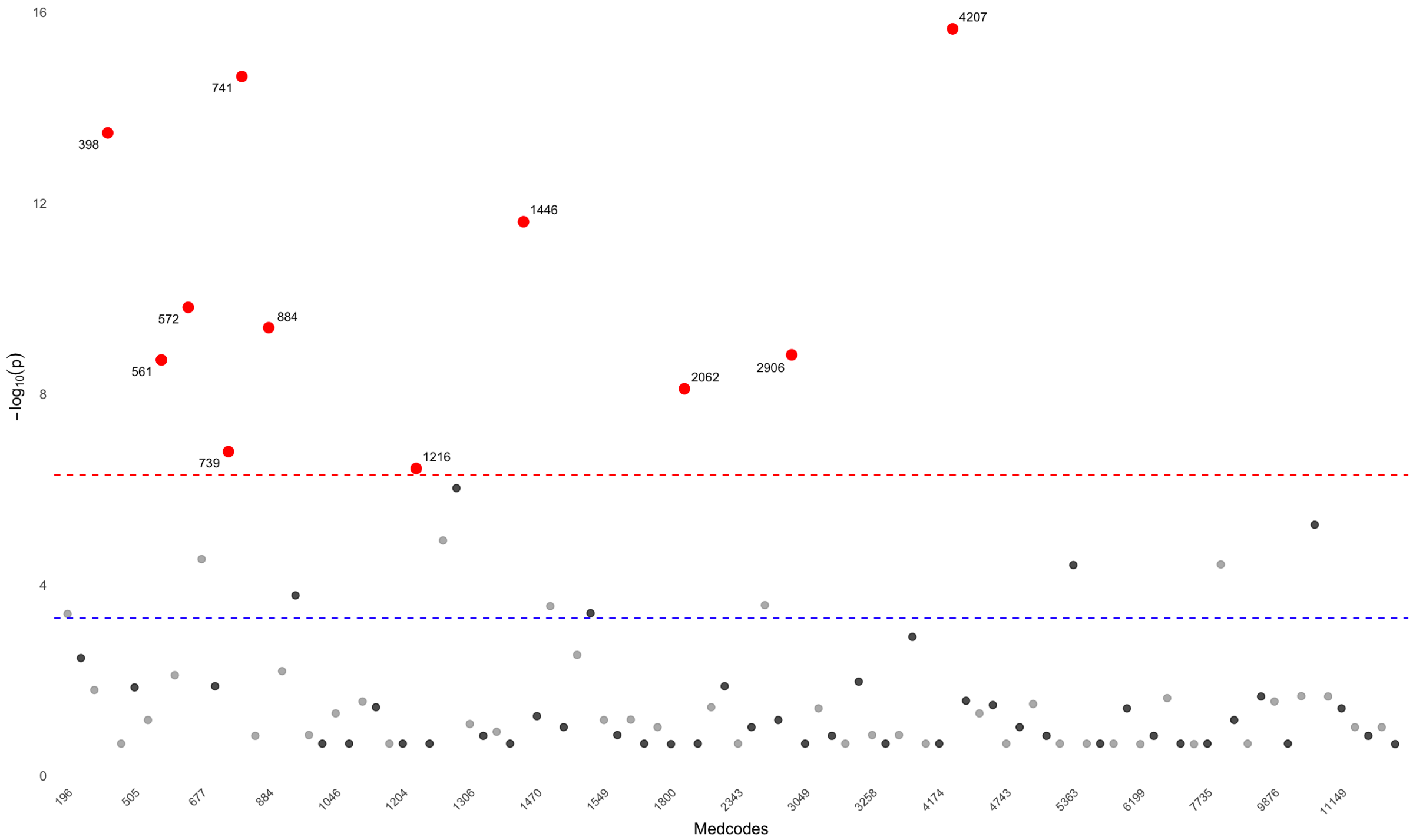


## F. 70 to 79 years

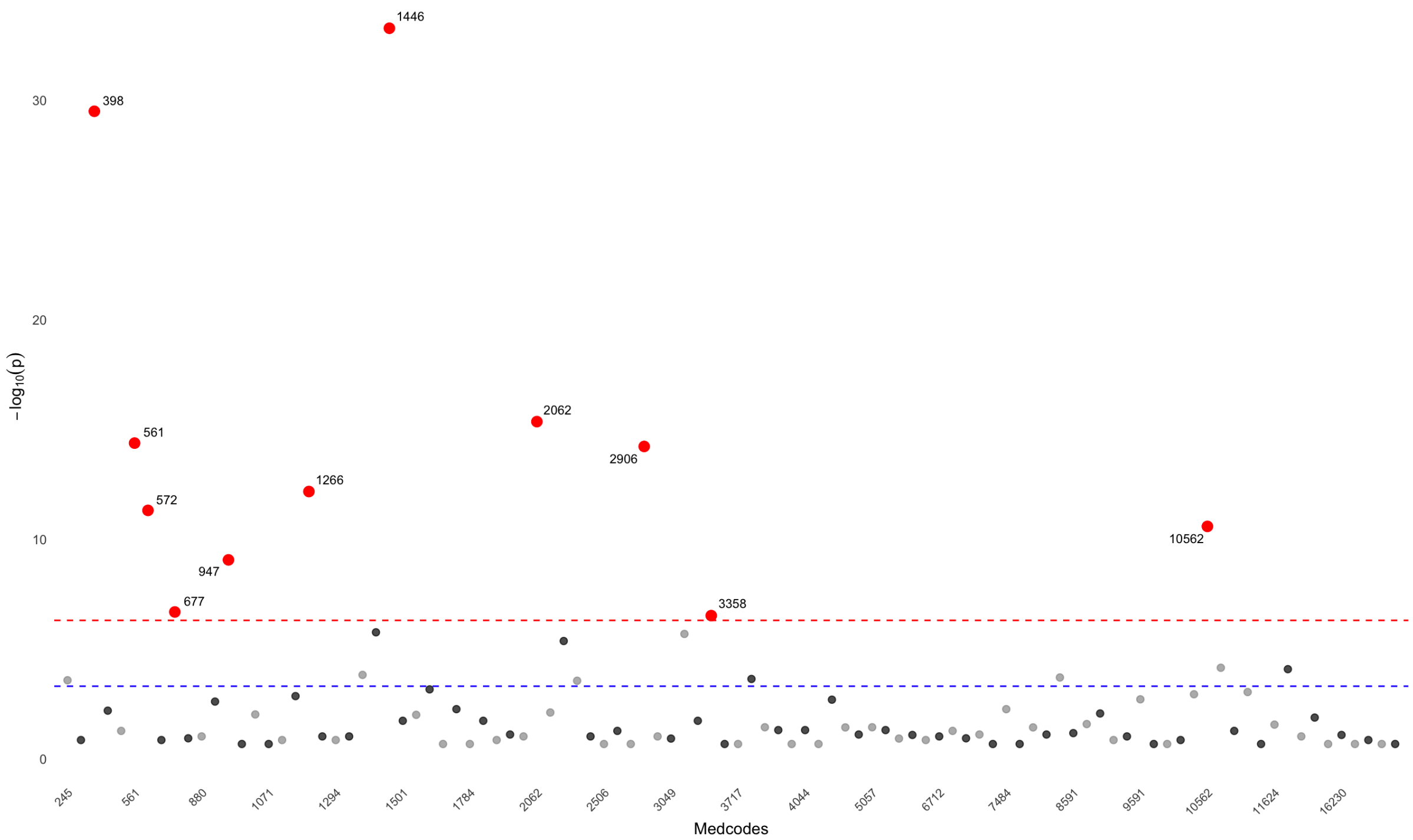

## G. 80 to 89 years

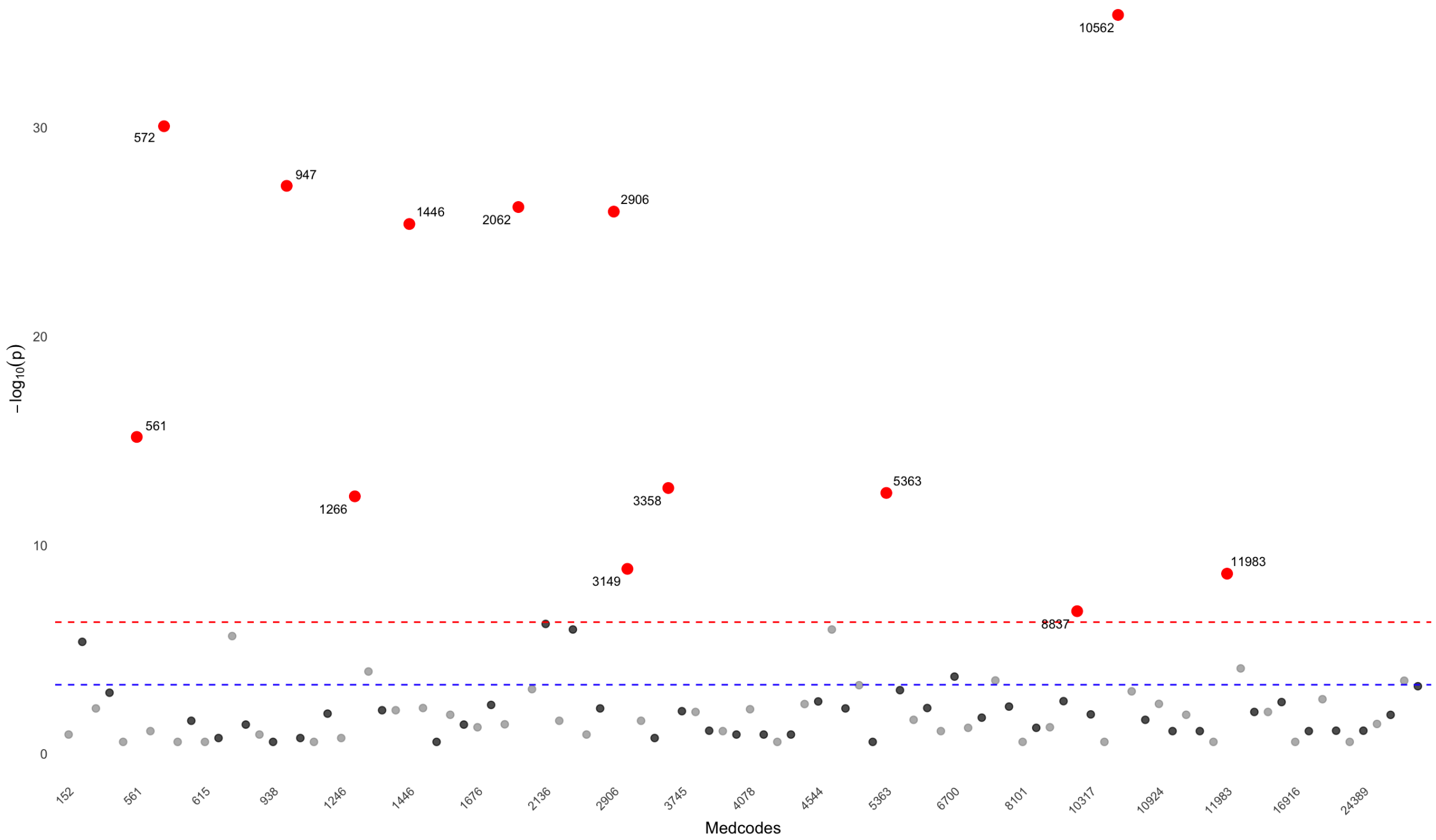


## H. ≥ 90 years

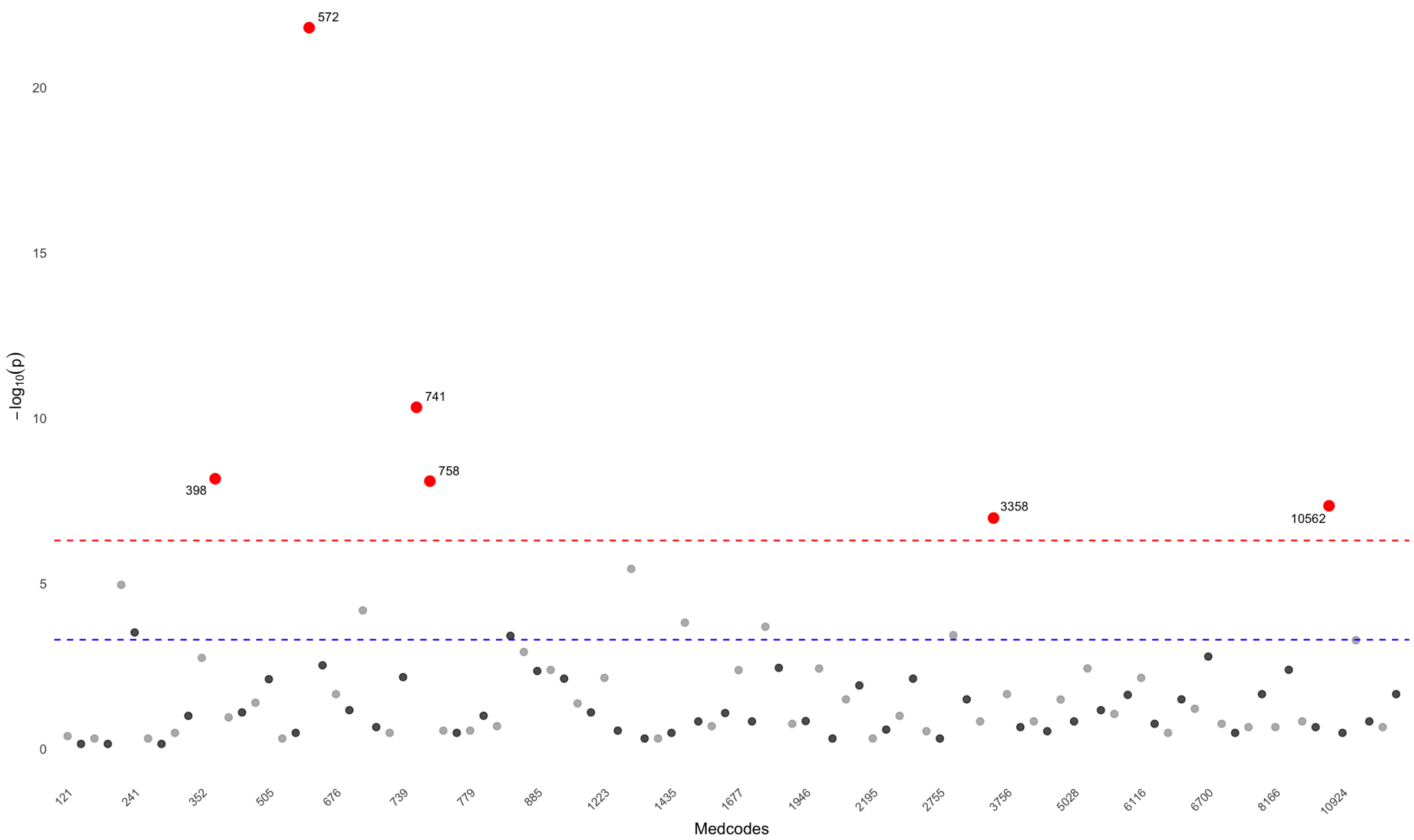

**Figure S-3.** Manhattan plots showing ICD-10 codes more frequently identified AF patients

## A. Men

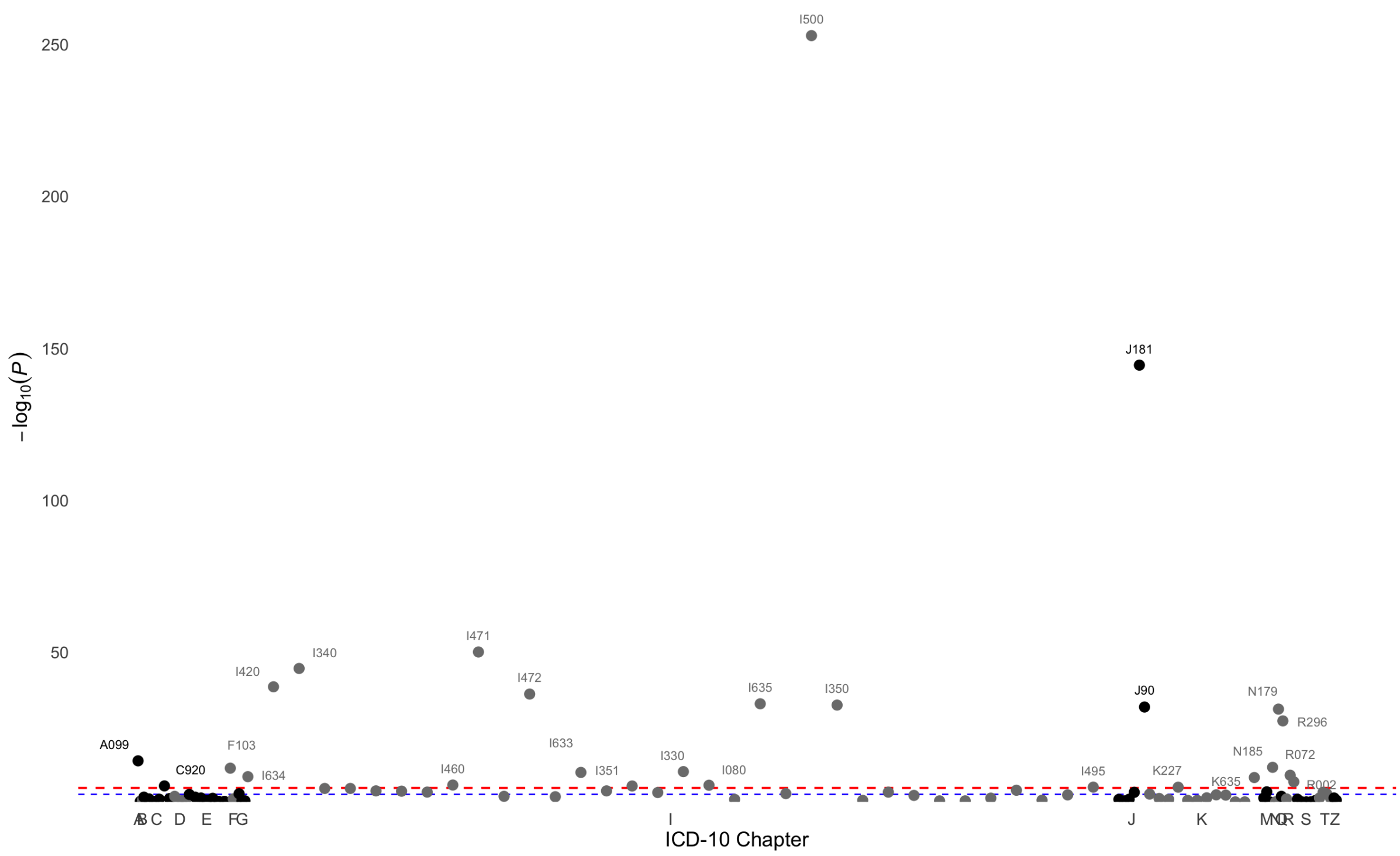


## B. Women

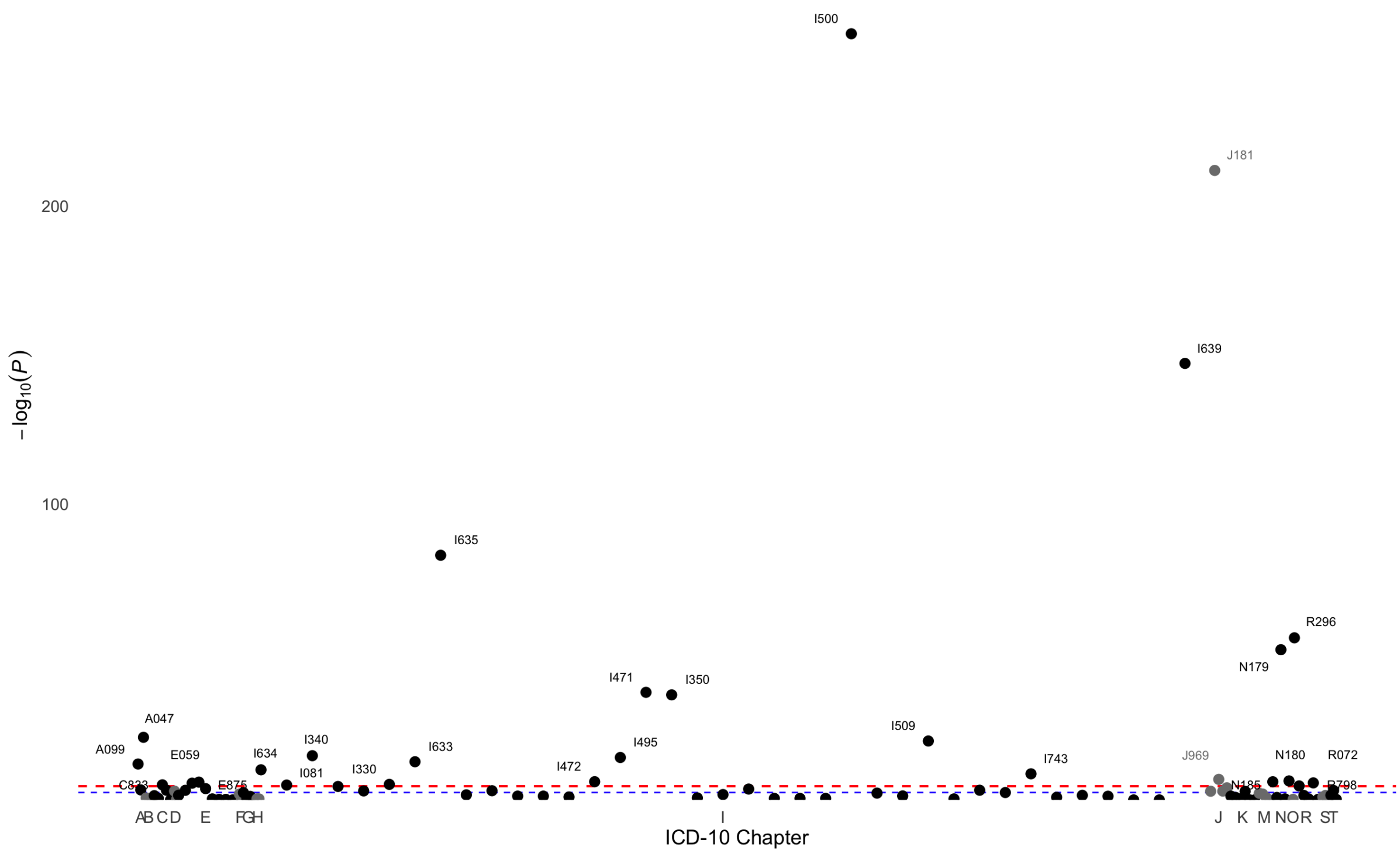

## C. <50 years

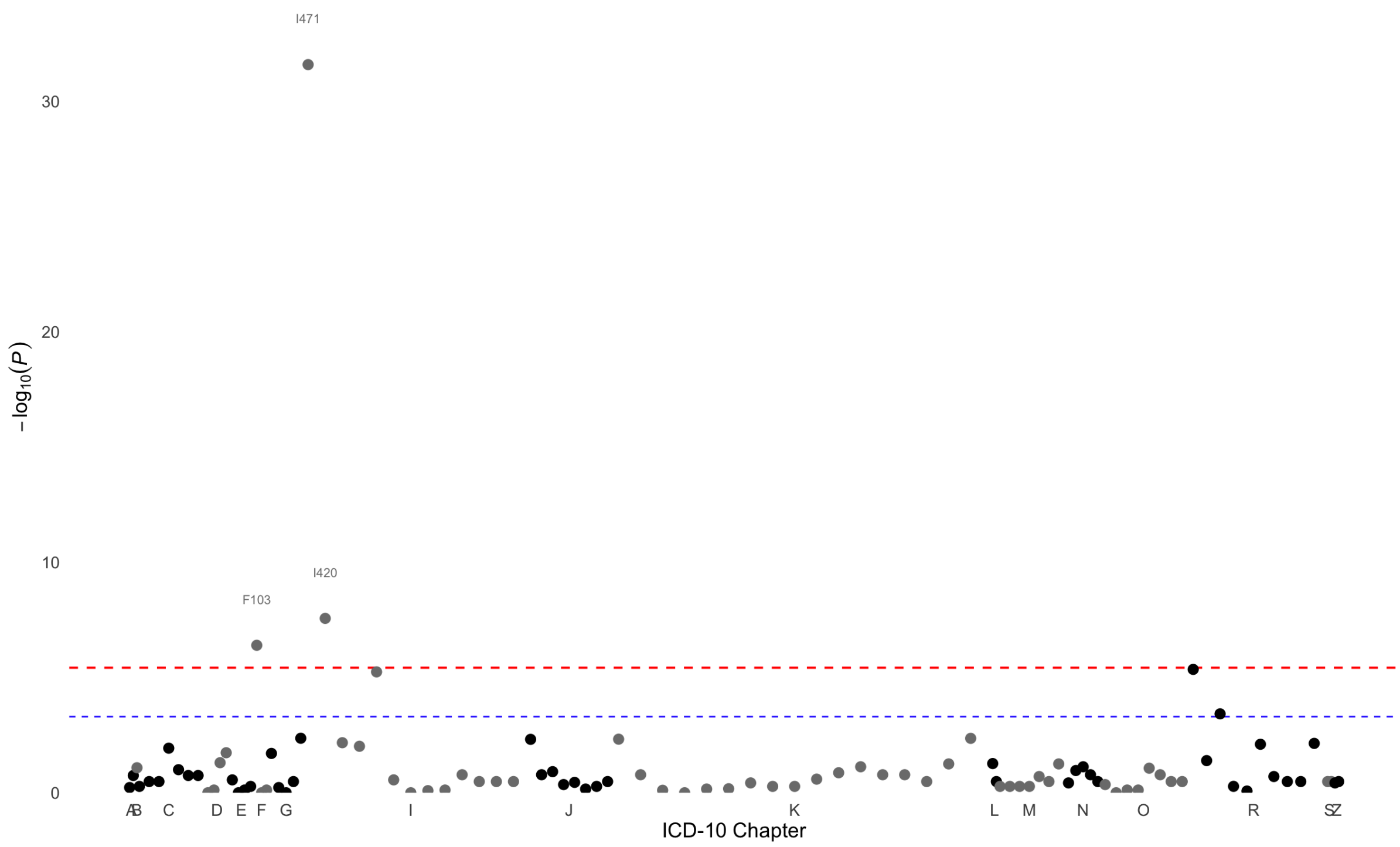


## D. 50 to 59 years

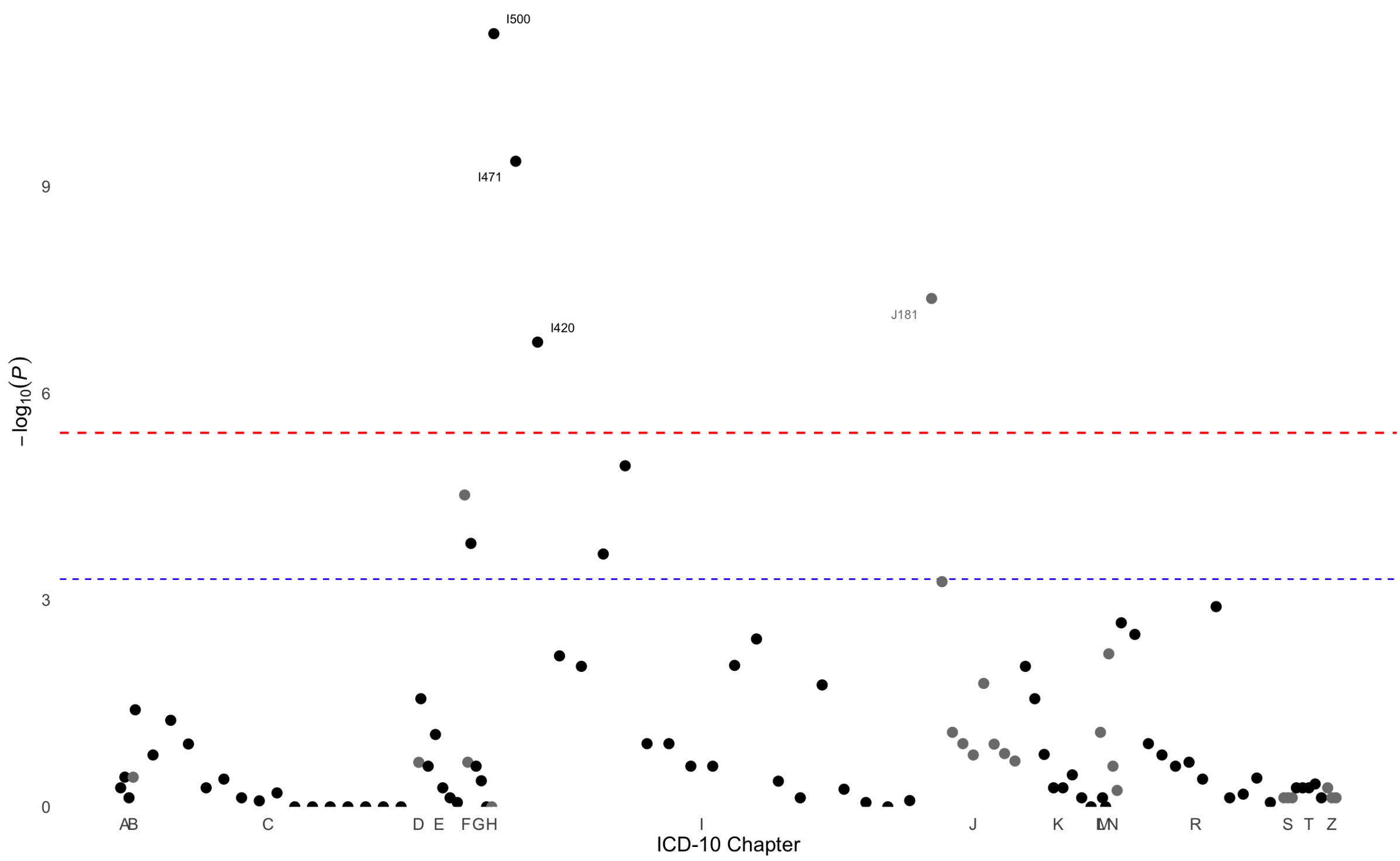

**E. 60 to 69 years**

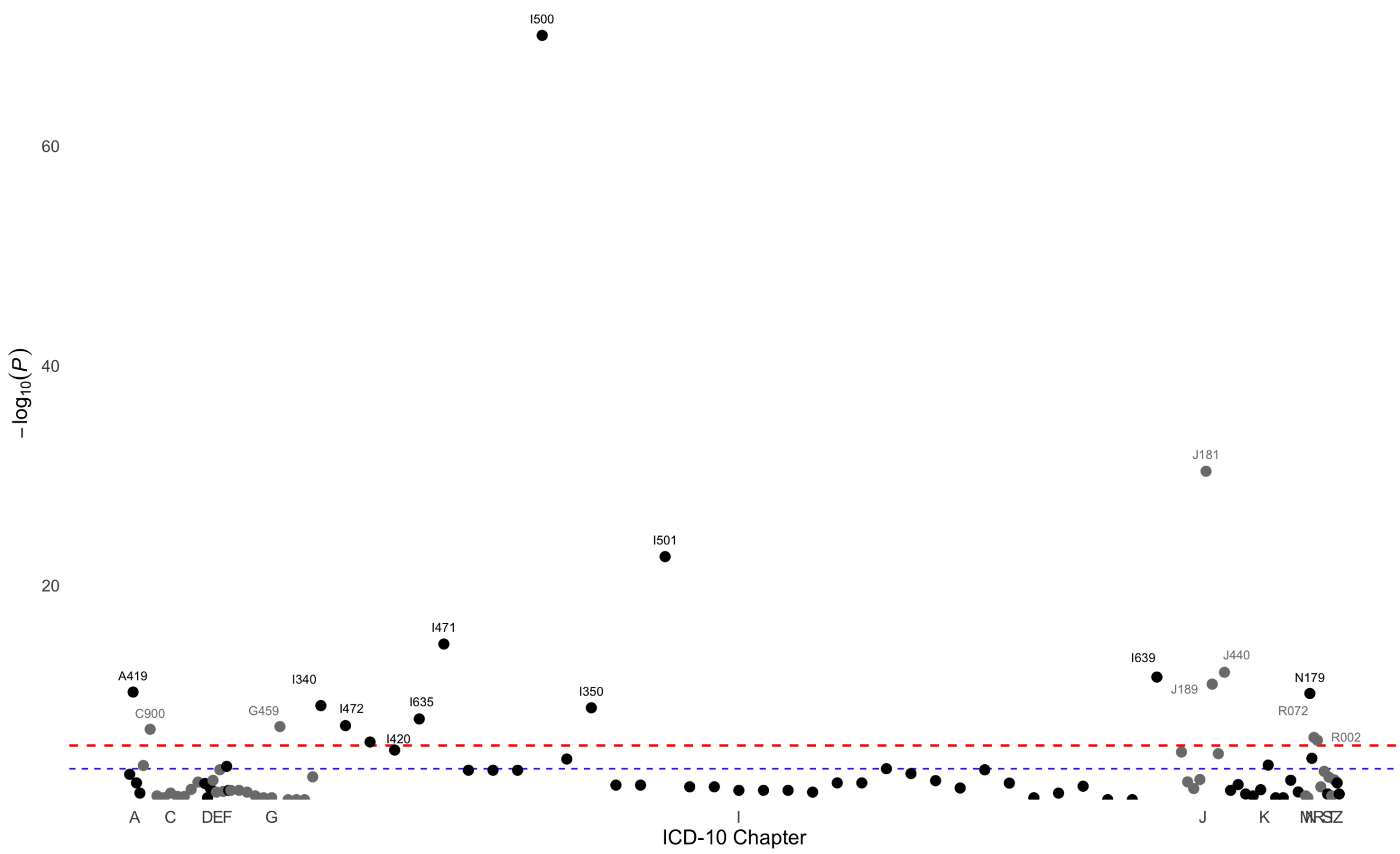


**F. 70 to 79 years**

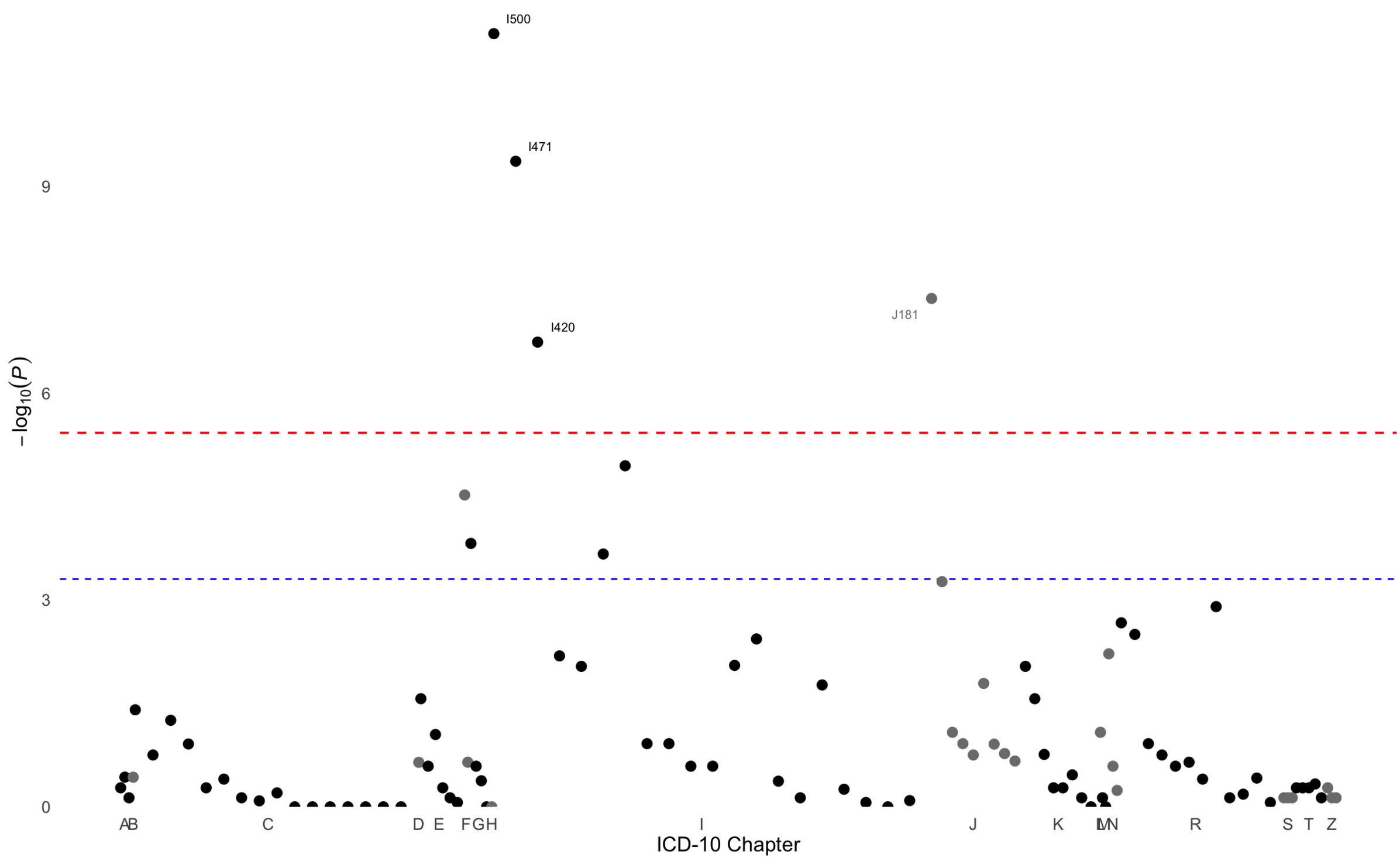

## G. 80 to 89 years

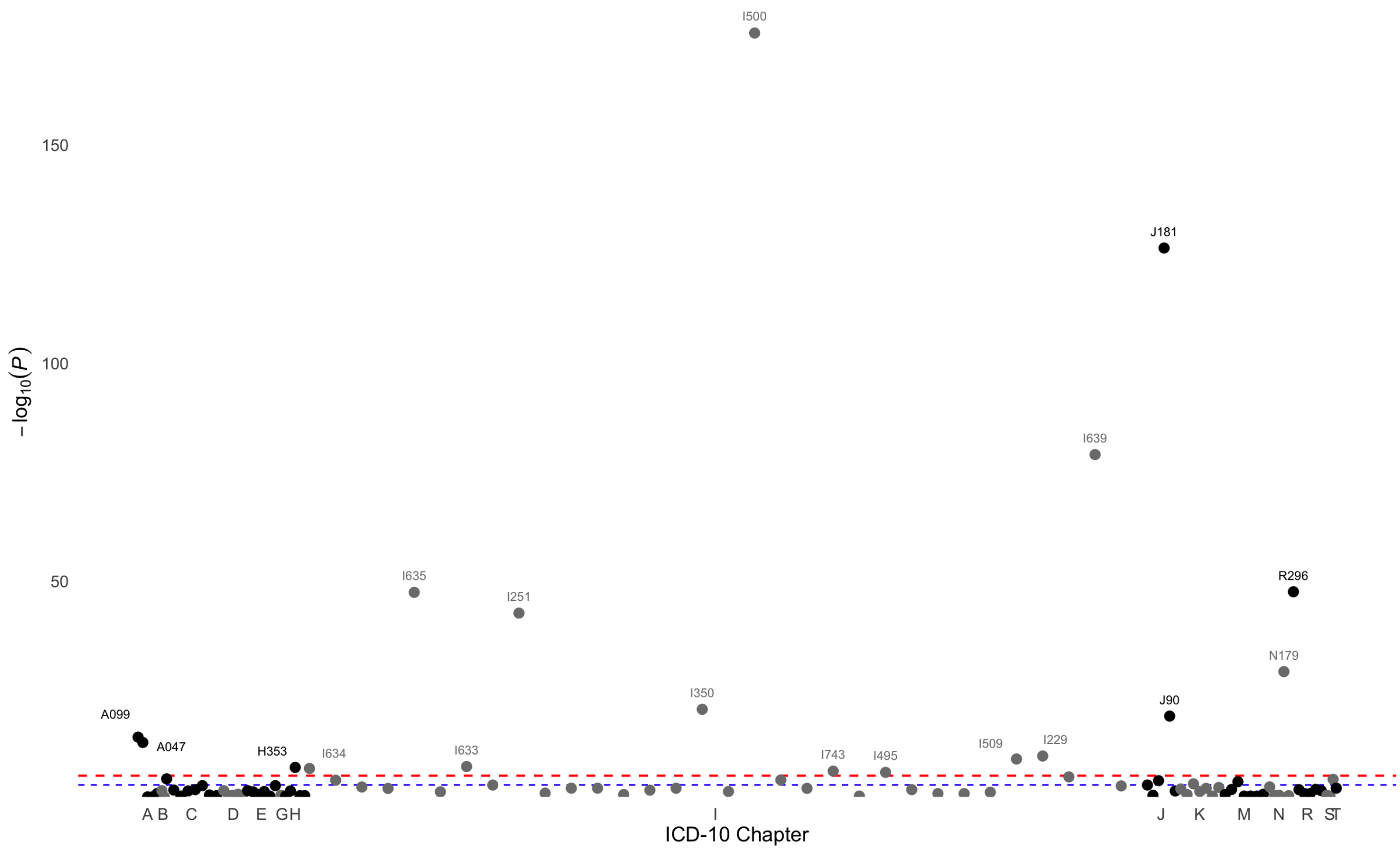


## H. ≥ 90 years

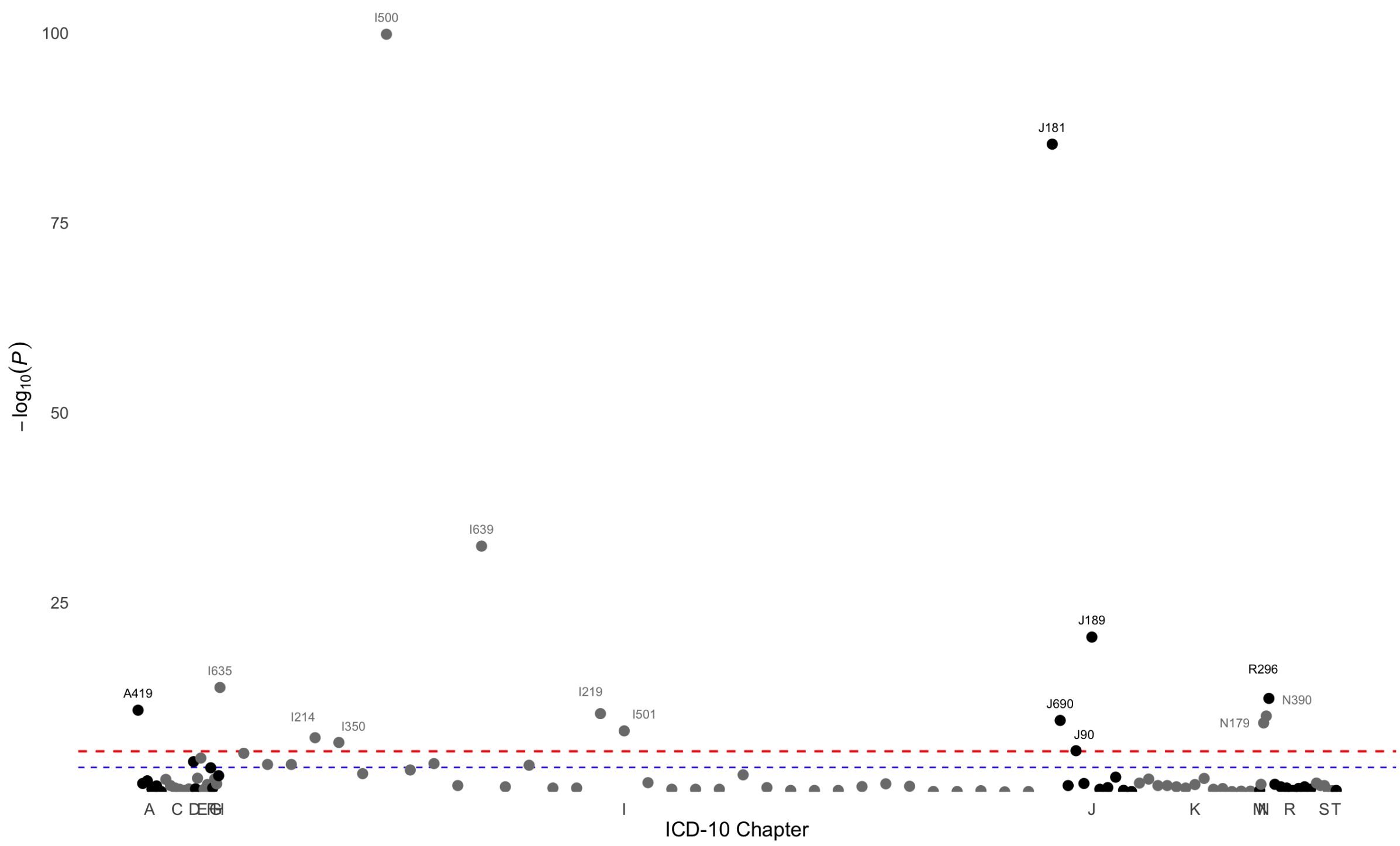

**Figure S.4.** Organ systems defined as ICD-10 chapters for the associations identified in the secondary analyses (AF without ischemic heart disease vs Ischemic heart disease without AF at baseline) per pre-defined AF patient stratum

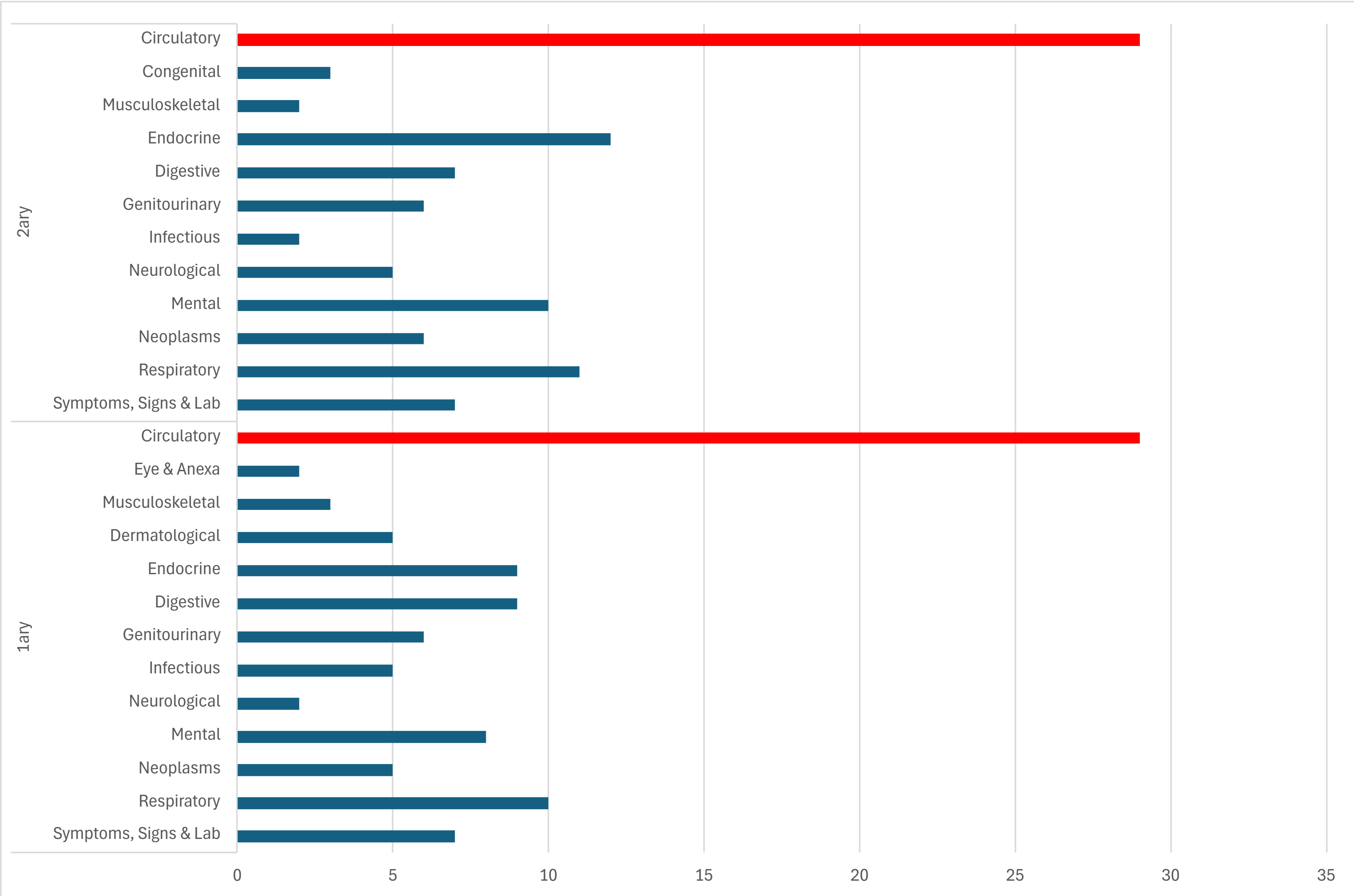

**Table S-1.** Utilized Electronic Health Record (EHR) codes

| | CPRD<br>Medical codes | HES<br>ICD-10 codes |
|---|---|---|
| **Atrial Fibrillation** | 1268, 1664, 1757, 2212, 3757, 6771, 18746, 23437, 35127, 39114, 45773, 57832, 63350, 90187, 90188, 90189, 90190, 90191, 96076, 96277 | I48 |
| **Ischemic heart disease** | 240, 241, 732, 733, 737, 1021, 1204, 1344, 1414, 1430, 1431, 1628, 1676, 1677, 1678, 1792, 2491, 3159, 3704, 4017, 4656, 5233, 5387, 5413, 5703, 5744, 6336, 7134, 7137, 7320, 7347, 7442, 7609, 7634, 7696, 7783, 8246, 8312, 8679, 8935, 8942, 9276, 9413, 9414, 9507, 9555, 10209, 10260, 10562, 11610, 11648, 11983, 12139, 12229, 12734, 12804, 13185, 13566, 13571, 14658, 14782, 14897, 14898, 15349, 15373, 15661, 15754, 16408, 17133, 17307, 17464, 17689, 17872, 18118, 18125, 18135, 18249, 18670, 18842, 18889, 19046, 19193, 19402, 19413, 19542, 19655, 19827, 20095, 20416, 20903, 21844, 22020, 22383, 22647, 22828, 23078, 23579, 23708, 23892, 24126, 24540, 24783, 25842, 26863, 26965, 26967, 26972, 26973, 26975, 27951, 27977, 28138, 28554, 28736, 28837, 29300, 29421, 29553, 29643, 29758, 29902, 30330, 30421, 31519, 31556, 32272, 32450, 32651, 32854, 33471, 33718, 33735, 34328, 34633, 34803, 34963, 35119, 35287, 35674, 35713, 36011, 36423, 36523, 37657, 37682, 37719, 38609, 38813, 39449, 39546, 39655, 39693, 40399, 40429, 41221, 41547, 41835, 42104, 42304, 42462, 42708, 43939, 44561, 45370, 45476, 45809, 45886, 45960, 46017, 46112, 46166, 46230, 46276, 47637, 48767, 50372, 51507, 51515, 52517, 52705, 54251, 54535, 55092, 55137, 55401, 55598, 56990, 57062, 57241, 58135, 59032, 59189, 59423, 59940, 60067, 60753, 61072, 61208, 61310, 61670, 62608, 62626, 63467, 64923, 66236, 66388, 66664, 66921, 67591, 67761, 68123, 68139, 68357, 68401, 68748, 69474, 69776, 70111, 70185, 70755, 72562, 72780, 85947, 86071, 86773, 87849, 92419, 92927, 93618, 93828, 95550, 96537, 96804, 96838 | I200, I201, I208, I209, I21, I22, I23, I241, I250, I251, I252, I253, I254, I255, I256, I258, I259 |
| **Pulmonary embolism** | 1266, 9701, 10280, 16976, 18121, 34687, 45740, 65459, 89238, 96209 | I26 |
| **Ischemic stroke** | 569, 3149, 5185, 5363, 5602, 6155, 8837, 9985, 10504, 15019, 15252, 16517, 23671, 24446, 25615, 26424, 27975, 33543, 34758, 36717, 40758, 47642, 53745, 57495, 91627, 94482 | I63.0, I63.1, I63.2, I63.3, I63.4, I63.5, I63.8, I63.9, I69.3 |
| **Major bleeding** | - | D62, D683, D698, D699, H113, H210, H313, H356, H431, H450, H922, I230, I312, I600, I601, I602, I603, I604, I605, I606, I607, I608, I609, I610, I611, I612, I613, I614, I615, I616, I618, I619, I620, I621, I629, I850, I983, J942, K226, K250, K252, K254, K256, K260, K262, K264, K266, K270, K272, K274, K276, K280, K282, K284, K286, K290, K625, K661, K762, K920, K921, K922, M250, N020, N021, N022, N023, N024, N025, N026, N027, N028, N029, N421, N920, N921, N923, N924, N930, N938, N939, N950, R040, R041, R042, R048, R049, R31, R58, S064, S065, S066, S260, S271, T792 |
| **Medication** | **British National Formulary (BNF) Product code** | |
| **Oral Anticoagulants** | 45, 61, 833, 1781, 4446, 5305, 6262, 8466, 8467, 13348, 13501, 13502, 13503, 13504, 13505, 13644, 15006, 15376, 17965, 23078, 30202, 30203, 31511, 31937, 33711, 34019, 34086, 34087, 34088, 34095, 34299, 34416, 34417, 34418, 34517, 34526, 34576, 34691, 34758, 34864, 34918, 36099, 38041, 38044, 39119, 39444, 39503, 39639, 39755, 39866, 40143, 42474, 43407, 43408, 43409, 43655, 44866, 46632, 46678, 46924, 47566, 47944, 48070, 48869, 48966, 50000, 51484, 51496, 51509, 53740, 53745, 53752, 54066, 54451, 54892, 54946, 55316, 55577, 56289, 56314, 56640, 57032, 58519, 58594, 58787, 58962, 59400, 59578, 60589, 60949, 62150, 62309, 62310, 63071, 64500, 64678, 65247, 65285, 65496, 65746, 65850, 65876, 66286, 66529, 66570, 68591, 68667, 68795, 69128, 70831, 71196, 71303, 71386, 72040, 72120, 39119, 39444, 39503, 39639, 39755, 42474, 46632, 46678, 47207, 47353, 47566, 47925, 48134, 48966, 53740, 54066, 54451, 56289, 56640, 58594, 62150, 64500, 64678, 65247, 65850, 65876, 66529, 72120 | |

**Table S-2.** Top reasons for primary care consultations in AF patients without ischemic heart (IHD) disease at baseline, compared to controls with IHD.

| Medcode | Term | Events AF | Events IHD | OR | L95%CI | U95%CI | P value |
|---|---|---|---|---|---|---|---|
| 7603 | Sleep apnoea | 479 | 9 | 53.39 | 27.61 | 103.24 | 7.09E-129 |
| 96919 | Colitis | 318 | 6 | 53.11 | 23.68 | 119.11 | 7.78E-86 |
| 3204 | Cardiomyopathy | 1723 | 33 | 52.81 | 37.42 | 74.53 | 8.97E-113 |
| 7535 | Primary dilated cardiomyopathy | 470 | 9 | 52.38 | 27.09 | 101.31 | 3.11E-126 |
| 9450 | Mitral valve regurgitation | 662 | 13 | 51.14 | 29.54 | 88.55 | 5.57E-177 |
| 5942 | Impaired left ventricular function | 1083 | 24 | 45.45 | 30.32 | 68.11 | 2.60E-285 |
| 5743 | Valvular heart disease | 316 | 7 | 45.24 | 21.39 | 95.67 | 6.96E-84 |
| 11828 | Vaginal thrush | 479 | 11 | 43.68 | 24.03 | 79.41 | 3.89E-126 |
| 8148 | Obstructive sleep apnoea | 907 | 21 | 43.45 | 28.19 | 66.97 | 7.90E-238 |
| 9286 | Tricuspid regurgitation, cause unspecified | 1014 | 24 | 42.53 | 28.37 | 63.76 | 4.09E-265 |
| 12550 | Left ventricular diastolic dysfunction | 290 | 7 | 41.51 | 19.61 | 87.85 | 2.65E-76 |
| 1294 | Mitral valve prolapse | 574 | 14 | 41.15 | 24.22 | 69.93 | 6.96E-150 |
| 4171 | [X]post-traumatic stress disorder | 245 | 6 | 40.9 | 18.2 | 91.93 | 1.68E-64 |
| 11761 | Scalp psoriasis | 447 | 11 | 40.75 | 22.41 | 74.13 | 8.29E-117 |
| 16496 | Injury to kidney | 317 | 8 | 39.71 | 19.68 | 80.09 | 7.25E-83 |
| 2520 | Pericardial effusion - acute | 276 | 7 | 39.5 | 18.65 | 83.64 | 3.13E-72 |
| 8966 | Left ventricular systolic dysfunction | 4093 | 108 | 38.93 | 32.16 | 47.14 | 2.35E-308 |
| 3903 | Malignant neoplasm of bronchus or lung NOS | 1465 | 38 | 38.92 | 28.2 | 53.72 | 7.06E-110 |
| 99526 | Barrett's oesophagus | 657 | 17 | 38.81 | 23.98 | 62.82 | 3.71E-170 |
| 104028 | Sepsis | 1040 | 27 | 38.78 | 26.46 | 56.83 | 8.89E-269 |

**Table S-3.** Top reasons for hospitalisation in AF patients without IHD at baseline, compared to controls with IHD.

| ICD10 code | Term | Events AF | Events IHD | OR | L95%CI | U95%CI | P value |
|---|---|---|---|---|---|---|---|
| R572 | Septic shock | 676 | 8 | 84.88 | 42.27 | 170.43 | 1.37E-188 |
| F171 | Mental and behavioural disorders due to use of tobacco | 17131 | 243 | 79.45 | 69.98 | 90.19 | 6.67E-995 |
| J151 | Pneumonia due to Pseudomonas | 464 | 6 | 77.57 | 34.67 | 173.56 | 6.80E-129 |
| I421 | Obstructive hypertrophic cardiomyopathy | 415 | 6 | 69.36 | 30.98 | 155.28 | 2.12E-114 |
| I151 | Hypertension secondary to other renal disorders | 402 | 6 | 67.18 | 30 | 150.43 | 1.46E-110 |
| Q211 | Atrial septal defect | 714 | 11 | 65.21 | 35.95 | 118.3 | 3.41E-195 |
| I518 | Other ill-defined heart diseases | 6067 | 101 | 62.56 | 51.38 | 76.16 | 6.39E-371 |
| I420 | Dilated cardiomyopathy | 2400 | 41 | 59.47 | 43.67 | 80.99 | 3.23E-148 |
| I429 | Cardiomyopathy, unspecified | 1632 | 28 | 58.92 | 40.55 | 85.6 | 1.81E-101 |
| F103 | Mental and behavioural disorders due to use of alcohol | 1336 | 23 | 58.6 | 38.8 | 88.5 | 1.98E-83 |
| C340 | Malignant neoplasm of unspecified main bronchus | 461 | 8 | 57.8 | 28.73 | 116.28 | 5.19E-125 |
| R458 | Other symptoms and signs involving emotional state | 903 | 16 | 56.77 | 34.63 | 93.08 | 1.34E-243 |
| I490 | Ventricular fibrillation and flutter | 1402 | 25 | 56.6 | 38.11 | 84.06 | 5.54E-89 |
| I341 | Mitral (valve) prolapse | 1273 | 23 | 55.81 | 36.95 | 84.3 | 1.92E-81 |
| E833 | Disorders of phosphorus metabolism and phosphatases | 492 | 9 | 54.84 | 28.36 | 106.04 | 1.08E-132 |
| N170 | Acute renal failure with tubular necrosis | 424 | 8 | 53.15 | 26.41 | 106.97 | 3.88E-114 |
| E668 | Other obesity | 1883 | 36 | 52.96 | 38.08 | 73.65 | 4.97E-123 |
| J432 | Centrilobular emphysema | 316 | 6 | 52.78 | 23.53 | 118.37 | 3.00E-85 |
| I313 | Pericardial effusion (noninflammatory) | 2733 | 53 | 52.5 | 40 | 68.91 | 3.27E-179 |
| I088 | Other multiple valve diseases | 626 | 12 | 52.38 | 29.59 | 92.74 | 8.16E-168 |
